# Supplementary material for: The effects of L-carnitine and fructose in improved Ham’s F10 on sperm culture in idiopathic severe asthenospermia within 24h
Source: PLoS One. 2025 Feb 10;20(2):e0306235. doi: 10.1371/journal.pone.0306235 (PMC11809793; doi:10.1371/journal.pone.0306235)
Supplement: S1 File — (DOCX) [file pone.0306235.s001.docx]

| a1: Percentage of forward motility of sperm (%) | | | | |  |  |
| --- | --- | --- | --- | --- | --- | --- |
| basic data 1 | NS1 | L4 | L8 | L12 | L16 | HF1 |
| 5.66 | 1.95 | 6.59 | 13.25 | 3.46 | 4.54 | 9.93 |
| 6.25 | 3.48 | 7.56 | 13.25 | 0.71 | 4.39 | 11.92 |
| 6.74 | 3.72 | 6.84 | 8.51 | 5.97 | 4 | 10.94 |
| 6.59 | 3.52 | 5.22 | 11.26 | 5.07 | 4.54 | 8.67 |
| 6.28 | 5.15 | 4.73 | 13.25 | 4.3 | 4.54 | 11.67 |
| 4.08 | 4.74 | 6.14 | 14.91 | 2.62 | 1.73 | 11.04 |
| 6.49 | 2.16 | 5.72 | 14.13 | 4.37 | 2.46 | 8.04 |
| 7.88 | 1.49 | 6.53 | 17.35 | 4.2 | 2.34 | 8.25 |
| 7.02 | 4.42 | 8.25 | 14.1 | 2.67 | 2.59 | 8.64 |
| 6.28 | 2.72 | 6.76 | 12.45 | 3.89 | 2.86 | 8.84 |
| 6.48 | 1.65 | 4.94 | 10.56 | 3.72 | 2.66 | 11.76 |
| 4.13 | 4.21 | 5 | 11.17 | 5.39 | 1.24 | 7.76 |
| 6.14 | 4.1 | 5.98 | 16.09 | 2.1 | 1.42 | 8.37 |
| 5.85 | 3.57 | 5.38 | 9.14 | 5.64 | 2.76 | 11.77 |
| 6.28 | 2.96 | 6.38 | 9.82 | 2.19 | 3.82 | 10.11 |
| 6.75 | 5.05 | 7.9 | 13.07 | 3.33 | 3.91 | 6.45 |
| 7.51 | 4.21 | 7.64 | 13.37 | 2.39 | 2.46 | 8.72 |
| 6.54 | 4.66 | 4.83 | 15.94 | 3.98 | 4.54 | 11.69 |
| 7.37 | 1.7 | 7.7 | 17.05 | 5.85 | 4.25 | 10.91 |
| 4.72 | 5.14 | 6.89 | 9.17 | 2.31 | 4.54 | 8.31 |
| 8.85 | 4.58 | 6.07 | 9.75 | 3.46 | 2.35 | 5.21 |
| 7.48 | 2.66 | 6.86 | 11.5 | 1.79 | 4.54 | 7.65 |
| 7.3 | 1.73 | 6.98 | 17.32 | 4.17 | 4.54 | 10.78 |
| 7.09 | 2.11 | 5.68 | 8.57 | 2.7 | 2.44 | 7.35 |
| 8.13 | 3.67 | 6.61 | 8.6 | 1.52 | 2.4 | 7.52 |
| 5.17 | 4.74 | 6.1 | 16.32 | 4.45 | 3.71 | 7.13 |
| 8.1 | 2.49 | 7.77 | 16.64 | 5.84 | 3.8 | 10.82 |
| 6.28 | 3.8 | 6.57 | 11.37 | 3.07 | 2.08 | 11.92 |
| 7.42 | 2.67 | 5.13 | 13.3 | 1.02 | 1.17 | 11.92 |
| 5.23 | 2.78 | 4.8 | 14.96 | 3.58 | 2.74 | 7.33 |
| 5.29 | 5.14 | 7.82 | 13.86 | 1.81 | 1.81 | 10.03 |
| 6.09 | 3.73 | 7.87 | 16.52 | 0.88 | 2.87 | 10.53 |
| 6.73 | 3.9 | 8.14 | 13.29 | 3.89 | 2.44 | 11.11 |
| 4.65 | 3.06 | 7.21 | 11.34 | 4.08 | 1.93 | 8.06 |
| 3.99 | 2.07 | 7.89 | 14.44 | 1.23 | 2.46 | 10.93 |
| 6.31 | 2.39 | 6.88 | 10.7 | 3.92 | 4.54 | 10.55 |
| 6.02 | 1.6 | 5.83 | 9.44 | 3.92 | 3.71 | 8.34 |
| 7.93 | 3.19 | 7.96 | 9.96 | 4.16 | 3.89 | 5.38 |
| 5.76 | 5.03 | 5.57 | 15.82 | 3.04 | 2.73 | 11.62 |
| 7.63 | 3.67 | 7.03 | 16.59 | 5.25 | 3.76 | 11.85 |
| 6.16 | 1.77 | 5.9 | 15.63 | 4.94 | 2.64 | 11.92 |
| 6.53 | 2.6 | 7.7 | 13.13 | 0.93 | 2.69 | 8.68 |
| 3.29 | 2.33 | 5.57 | 14.87 | 2.69 | 1.65 | 7.22 |
| 6.09 | 2.71 | 6.31 | 14.5 | 5.71 | 2.24 | 7.22 |
| 5.66 | 2.47 | 4.95 | 16.66 | 4.07 | 4.22 | 11.92 |
| 5.24 | 4.85 | 8.21 | 15.8 | 5.55 | 1.63 | 8.55 |
| 5.13 | 3.67 | 4.99 | 9.6 | 4.46 | 4.07 | 10.01 |
| 8.25 | 2.57 | 6.11 | 10.87 | 5.68 | 4.54 | 8.75 |
| 8.4 | 4.7 | 5.3 | 17.35 | 5.27 | 4.54 | 7.1 |
| 4.37 | 2.97 | 6.46 | 11.39 | 1.54 | 2.8 | 11.15 |
| 4.91 | 3.29 | 7.35 | 13.1 | 1.26 | 1.53 | 6.63 |
| 4.28 | 2.41 | 4.74 | 9.2 | 5.45 | 4.54 | 11.72 |
| 5.23 | 2.81 | 7.18 | 16.63 | 5.76 | 3.88 | 6.78 |
| 7.11 | 2.73 | 5.34 | 17.11 | 4.68 | 4.54 | 7.46 |
| 6.28 | 1.97 | 7.69 | 16 | 4.55 | 2.2 | 8.76 |
| 5.53 | 3.94 | 7.54 | 10.89 | 3.77 | 3.8 | 9.99 |
| 6.84 | 3.22 | 7.64 | 16.32 | 5.05 | 4.06 | 10.23 |
| 6.43 | 3.79 | 4.86 | 14.98 | 1.64 | 2.64 | 8.55 |
| 6.99 | 4.16 | 4.91 | 13.61 | 5.77 | 1.05 | 5.13 |
| 7.29 | 4.74 | 7.01 | 15.77 | 3.28 | 4.54 | 11.92 |
| 5.54 | 4.45 | 7.04 | 8.9 | 5.2 | 5.56 | 8.04 |

| a2: Percentage of non-forward motility of sperm (%) | | | | |  |  |
| --- | --- | --- | --- | --- | --- | --- |
| basic data 1 | NS1 | L4 | L8 | L12 | L16 | HF1 |
| 6.23 | 4.42 | 3.52 | 8.9 | 3.24 | 5.56 | 7.15 |
| 5.61 | 0.55 | 6.86 | 9.18 | 0.77 | 5.56 | 2.25 |
| 3.61 | 3.02 | 3.96 | 5.02 | 0.21 | 0.26 | 5.85 |
| 4.04 | 4.34 | 3.37 | 6.19 | 0.56 | 0.26 | 6.06 |
| 5.11 | 0.6 | 6.4 | 7.78 | 0.21 | 0.28 | 7.38 |
| 1.43 | 0.8 | 5.44 | 8.48 | 0.31 | 4.94 | 2.82 |
| 3.58 | 2.63 | 5.75 | 8.14 | 3.02 | 0.71 | 4.04 |
| 4.97 | 4.15 | 5.9 | 4.44 | 0.65 | 5.51 | 6.29 |
| 4.01 | 0.97 | 3.09 | 6.15 | 1.32 | 5.56 | 4.45 |
| 5.84 | 1.7 | 3.09 | 8.55 | 2.78 | 3.96 | 6.61 |
| 2.55 | 2.36 | 3.87 | 8.87 | 2.75 | 4.82 | 7 |
| 5.33 | 4.45 | 3.4 | 7.55 | 4.29 | 5.01 | 3.17 |
| 7.48 | 2.96 | 5.3 | 4.68 | 1.08 | 3.73 | 6.99 |
| 3.57 | 1.31 | 6.76 | 8.08 | 2.8 | 0.26 | 4.81 |
| 2.7 | 3.89 | 3.09 | 7.7 | 5.2 | 3.91 | 6.45 |
| 4.22 | 1.21 | 4.26 | 5.92 | 0.35 | 4.86 | 6.97 |
| 5.62 | 3.55 | 3.22 | 9.09 | 0.21 | 4.88 | 5.92 |
| 3.07 | 3.56 | 3.22 | 5.58 | 0.23 | 5.56 | 6.53 |
| 5.76 | 2.75 | 5.53 | 6.86 | 1.13 | 1.84 | 7.25 |
| 4.71 | 0.77 | 3.09 | 6.25 | 4.08 | 4.11 | 3.49 |
| 6.17 | 1.38 | 6.86 | 5.76 | 4.35 | 1.81 | 4.55 |
| 5.65 | 2.09 | 5.92 | 9.51 | 0.58 | 1.6 | 4.26 |
| 5.62 | 3.28 | 5.73 | 9.48 | 0.21 | 1.12 | 4.64 |
| 5.61 | 0.59 | 3.6 | 5.72 | 4.13 | 5.2 | 3.3 |
| 5.06 | 4.06 | 4.42 | 9.81 | 1.19 | 5.56 | 3.39 |
| 5.7 | 2.98 | 5.97 | 9.8 | 3.16 | 4.08 | 7.45 |
| 5.49 | 0.63 | 4.34 | 8.26 | 0.21 | 0.26 | 4.8 |
| 2.95 | 4.21 | 6.89 | 6.16 | 3.9 | 1.21 | 4.74 |
| 5.06 | 1.28 | 3.09 | 6.5 | 5.2 | 4.69 | 6.66 |
| 7.54 | 1.61 | 5.78 | 8.2 | 3.2 | 4.11 | 3.02 |
| 5.08 | 3.59 | 6.24 | 10.73 | 3.5 | 4.05 | 6.92 |
| 3.43 | 1.79 | 6.65 | 9.15 | 1.27 | 4.73 | 2.27 |
| 5.06 | 0.74 | 4.24 | 5.78 | 0.21 | 1.07 | 5.86 |
| 5.92 | 1.8 | 6.11 | 8.95 | 4.44 | 0.26 | 6.88 |
| 5.36 | 1.71 | 4.19 | 4.9 | 5.18 | 4.85 | 4.33 |
| 3.94 | 1.98 | 3.98 | 5.96 | 0.21 | 1.24 | 6.98 |
| 3.46 | 2.06 | 5.44 | 6.55 | 0.21 | 0.8 | 4.13 |
| 5.79 | 2.66 | 6.94 | 8.57 | 2.91 | 5.08 | 3.08 |
| 5.5 | 3.19 | 6.38 | 4.13 | 3.68 | 5.56 | 4.08 |
| 6.33 | 1.51 | 6.05 | 9.49 | 0.21 | 0.26 | 6.74 |
| 4.36 | 0.74 | 4.45 | 8.7 | 0.21 | 3.76 | 5.75 |
| 3.78 | 1.04 | 6.3 | 4.11 | 3.52 | 0.6 | 3.58 |
| 5.31 | 0.85 | 3.3 | 8.29 | 1.02 | 1.48 | 7.39 |
| 2.95 | 4.11 | 6.81 | 5.16 | 0.69 | 0.29 | 6.94 |
| 6.02 | 4.37 | 3.09 | 4.16 | 2.77 | 0.26 | 7.53 |
| 6.36 | 1.97 | 5.79 | 8.02 | 0.28 | 5.56 | 6.48 |
| 2.95 | 3.56 | 5.84 | 5.45 | 2.68 | 4.52 | 7.39 |
| 5.45 | 0.55 | 6.15 | 8.79 | 4.01 | 0.26 | 6.32 |
| 5.47 | 3.21 | 6.27 | 5.99 | 0.21 | 1.53 | 7.8 |
| 6.36 | 3.72 | 3.09 | 11.42 | 0.21 | 1.37 | 6.56 |
| 3.9 | 1.53 | 5.27 | 8.17 | 0.5 | 5.08 | 4.41 |
| 4.47 | 1.37 | 4.04 | 4.96 | 1.14 | 4.71 | 6.54 |
| 4.86 | 2.61 | 4.53 | 4.18 | 5.09 | 1.3 | 4.44 |
| 5.4 | 3.9 | 6.68 | 7.76 | 0.96 | 4.11 | 4.4 |
| 4.61 | 4.07 | 4.42 | 5.9 | 3.82 | 1.35 | 4.46 |
| 3.48 | 3.56 | 5.6 | 8.8 | 2.92 | 1.51 | 4.23 |
| 1.03 | 2.02 | 5.45 | 9.17 | 0.21 | 1.21 | 3.82 |
| 6.42 | 0.58 | 3.22 | 8.32 | 3.91 | 1.05 | 2.8 |
| 5.41 | 0.55 | 3.09 | 7.03 | 0.2 | 0.26 | 2.25 |
| 5.54 | 4.45 | 7.04 | 8.9 | 5.2 | 5.56 | 8.04 |
