# Supplementary material for: The effects of L-carnitine and fructose in improved Ham’s F10 on sperm culture in idiopathic severe asthenospermia within 24h
Source: PLoS One. 2025 Feb 10;20(2):e0306235. doi: 10.1371/journal.pone.0306235 (PMC11809793; doi:10.1371/journal.pone.0306235)
Supplement: S2 File — (DOCX) [file pone.0306235.s002.docx]

b1: Percentage of forward motility of sperm (%)

| basic data 2+A2:G54 | NS2 | F0.125 | F0.250 | F0.375 | F0.500 | HF2 |
| --- | --- | --- | --- | --- | --- | --- |
| 7.08 | 4.28 | 5.99 | 7.52 | 12.06 | 9.91 | 5.52 |
| 7.98 | 3.48 | 5 | 8.61 | 12.06 | 6.11 | 8.87 |
| 6.54 | 2.18 | 6.04 | 6.4 | 7.18 | 8.15 | 5.16 |
| 5.43 | 2.78 | 5.04 | 5.75 | 11.88 | 7.66 | 4.85 |
| 6.55 | 4.56 | 4.66 | 5.53 | 7.24 | 5.99 | 8.87 |
| 4.59 | 2.34 | 8 | 8.29 | 7.03 | 9.55 | 8.87 |
| 6.54 | 1.03 | 5.09 | 5.92 | 7.65 | 6.73 | 8.87 |
| 5.71 | 2.31 | 8 | 5.53 | 12.06 | 7.15 | 8.87 |
| 7.37 | 4.36 | 4.02 | 8.65 | 5.91 | 6.26 | 5.63 |
| 6.76 | 4.71 | 8 | 6.17 | 7.04 | 10.25 | 8.87 |
| 6.38 | 2.74 | 5.77 | 7.95 | 12.06 | 9.09 | 5.39 |
| 6.08 | 2.86 | 5.76 | 7.66 | 8.32 | 9.11 | 4.13 |
| 5.6 | 4.45 | 5.48 | 6.13 | 11.46 | 9.93 | 8.87 |
| 4.85 | 1.73 | 8 | 5.75 | 6.98 | 7.18 | 8.87 |
| 6.06 | 2.4 | 5.58 | 5.64 | 12.06 | 7.17 | 4.87 |
| 6.88 | 5.84 | 8 | 6.39 | 6.08 | 8.12 | 6.98 |
| 9.1 | 3.97 | 7.91 | 8.01 | 6.84 | 9.53 | 8.87 |
| 7.15 | 3.52 | 6.01 | 6.56 | 7.9 | 10.25 | 8.63 |
| 6.54 | 5.14 | 7.93 | 5.53 | 11.02 | 9.4 | 8.87 |
| 6.9 | 3.07 | 7.62 | 8.85 | 10.75 | 9.08 | 8.87 |
| 5.9 | 6 | 5.66 | 8.49 | 10.96 | 10.25 | 8.87 |
| 5.02 | 3.2 | 4.98 | 7.54 | 8.36 | 9.3 | 8.76 |
| 3.39 | 2.91 | 8 | 5.53 | 6.43 | 9.3 | 5.42 |
| 6.33 | 4.82 | 8 | 6.13 | 7.85 | 9.13 | 6 |
| 8.1 | 3.47 | 3.78 | 7.64 | 6.67 | 5.97 | 4.29 |
| 5.4 | 3.6 | 8 | 7.74 | 12.06 | 7.58 | 8.87 |
| 6.69 | 4.15 | 5.55 | 6.21 | 10.86 | 9.39 | 5.97 |
| 4.04 | 1.33 | 4.26 | 6.38 | 11.74 | 7.14 | 8.56 |
| 6.99 | 1.57 | 8 | 8.06 | 7.59 | 7.57 | 5.47 |
| 3.44 | 3.02 | 7.9 | 8.85 | 4.94 | 8.94 | 4.78 |
| 6.31 | 3.22 | 8 | 6.42 | 6.46 | 7.37 | 8.87 |
| 3.81 | 2.91 | 8 | 6.23 | 10.89 | 7.9 | 8.87 |
| 6.87 | 4.57 | 5.38 | 6.35 | 7.49 | 9.21 | 8.87 |
| 9.77 | 2.36 | 8 | 8.25 | 10.31 | 9.64 | 5.44 |
| 6.48 | 2.11 | 7.76 | 8.01 | 11.47 | 9.83 | 4.64 |
| 7.75 | 4.4 | 4.37 | 6.41 | 6.9 | 9.44 | 8.87 |
| 6.54 | 4.22 | 5.45 | 7.61 | 7.8 | 9.18 | 8.21 |
| 5.56 | 4.51 | 8 | 5.98 | 11.62 | 7.58 | 4.13 |
| 6.54 | 1.7 | 7.94 | 7.71 | 7.7 | 9.72 | 5.66 |
| 7.98 | 2.67 | 4.5 | 5.81 | 12.06 | 7.74 | 4.46 |
| 8.07 | 4.69 | 5.51 | 7.49 | 8.19 | 7.02 | 8.87 |
| 7.1 | 2.98 | 6 | 6.23 | 8.41 | 9.94 | 8.87 |
| 5.4 | 1.82 | 8 | 6.52 | 12.06 | 10.25 | 8.87 |
| 6.42 | 3.62 | 8 | 7.88 | 8.08 | 10.25 | 4.36 |
| 5.64 | 3.57 | 8 | 8.02 | 7.22 | 9.59 | 8.62 |
| 8.22 | 3.18 | 5.91 | 5.65 | 12.06 | 9.29 | 4.13 |
| 8.35 | 6 | 7.9 | 7.59 | 8.15 | 7.47 | 8.43 |
| 8.36 | 2.32 | 8 | 8.42 | 7.45 | 10.06 | 4.78 |
| 6.54 | 4.35 | 5.78 | 5.71 | 8.08 | 7.3 | 5.2 |
| 8.61 | 2.02 | 7.82 | 5.87 | 11.26 | 7.9 | 8.8 |
| 5.15 | 4.46 | 7.87 | 7.83 | 11.96 | 8.1 | 8.63 |
| 7.08 | 3.74 | 4.47 | 8.33 | 7.09 | 10.23 | 5.51 |
| 5.81 | 2.71 | 8 | 6.48 | 12.01 | 8.99 | 5.82 |
| 6.99 | 2.09 | 4.05 | 7.79 | 10.42 | 9.61 | 8.87 |
| 4.33 | 2.39 | 5.88 | 5.75 | 5.17 | 9.61 | 8.87 |
| 8.73 | 3.54 | 8 | 8.76 | 12.06 | 9.12 | 4.18 |
| 7.38 | 2.99 | 8 | 8.85 | 11.58 | 7.98 | 4.13 |
| 6.71 | 3.63 | 4.47 | 5.79 | 7.32 | 9.85 | 4.83 |
| 6.37 | 0.72 | 3.62 | 5.53 | 4.94 | 5.9 | 4.13 |
| 8.34 | 6 | 8 | 8.85 | 12.06 | 10.25 | 8.87 |

b2: Percentage of non-forward motility of sperm (%)

| basic data 2 | NS2 | F0.125 | F0.250 | F0.375 | F0.500 | HF2 |
| --- | --- | --- | --- | --- | --- | --- |
| 5.21 | 1.32 | 2.61 | 5.16 | 5.38 | 3.9 | 6.98 |
| 0.51 | 1.2 | 4.02 | 3.12 | 7.92 | 9.83 | 4.09 |
| 3.25 | 3.14 | 4.27 | 5.96 | 6.09 | 4.82 | 6.37 |
| 5.23 | 1.33 | 4.05 | 4.03 | 8.9 | 8.1 | 3.54 |
| 4.69 | 3.83 | 3.07 | 5.08 | 8.32 | 9.5 | 6.16 |
| 4.91 | 1.57 | 6.49 | 5.09 | 9 | 4.46 | 3.54 |
| 4.88 | 0.76 | 6.01 | 4.42 | 5.65 | 5.9 | 4.15 |
| 4.96 | 3.1 | 5.83 | 2.66 | 5.93 | 6.24 | 4.34 |
| 3.51 | 2.72 | 4.17 | 3.87 | 4.88 | 8.28 | 3.54 |
| 5.8 | 1.64 | 4.57 | 4.4 | 7.36 | 7.48 | 5.72 |
| 5.73 | 3.34 | 5.44 | 7.07 | 4.88 | 9.79 | 4.04 |
| 3.15 | 0.62 | 5.92 | 6.57 | 6.05 | 5.83 | 7.17 |
| 4.49 | 2.71 | 5.25 | 4.52 | 4.88 | 4.12 | 3.58 |
| 6.25 | 2.77 | 5.77 | 6.45 | 7.29 | 4.88 | 4.12 |
| 4.98 | 3.03 | 3.83 | 5.45 | 5.46 | 8.36 | 4.15 |
| 3.54 | 3.4 | 4.32 | 6.9 | 7.98 | 5.85 | 6.21 |
| 6.49 | 3.04 | 6.83 | 3.58 | 5.2 | 3.71 | 3.8 |
| 5.73 | 2.95 | 6.36 | 5.98 | 6.06 | 3.71 | 6.22 |
| 4.98 | 2.98 | 5.9 | 3.89 | 8.11 | 10.03 | 3.88 |
| 5.38 | 2.77 | 5.78 | 7.07 | 5.32 | 8.1 | 6.39 |
| 5.69 | 0.78 | 5.39 | 6.84 | 4.88 | 8 | 6.73 |
| 5.5 | 0.88 | 4.28 | 4.17 | 7.14 | 9.19 | 6.45 |
| 5.9 | 1.66 | 4.17 | 5.09 | 7.17 | 6.35 | 3.88 |
| 5.27 | 2.75 | 6.83 | 5.61 | 5.3 | 7.14 | 4.36 |
| 5.02 | 1.4 | 6.83 | 6.87 | 5.42 | 5.7 | 7.18 |
| 4.95 | 0.35 | 2.61 | 5.32 | 8.79 | 4.33 | 6.42 |
| 5.36 | 1.08 | 2.83 | 2.73 | 7.35 | 5.01 | 3.74 |
| 6.64 | 0.35 | 4.1 | 4.1 | 4.91 | 5.36 | 3.63 |
| 4.1 | 0.41 | 5.51 | 3.68 | 9.11 | 9.32 | 5.98 |
| 4.87 | 3.35 | 4.43 | 6.34 | 4.88 | 8.45 | 5.9 |
| 3.88 | 2.58 | 5.4 | 3.13 | 5.18 | 7.18 | 6.14 |
| 5.45 | 1.16 | 3.62 | 2.18 | 4.99 | 7.43 | 7.18 |
| 5.16 | 0.96 | 6.82 | 4.46 | 6.15 | 4.71 | 6.45 |
| 3.21 | 3.29 | 3.16 | 3.32 | 8.35 | 8.37 | 3.81 |
| 5.98 | 0.46 | 3.69 | 5.5 | 5.79 | 4.22 | 3.8 |
| 4.64 | 1.2 | 3.51 | 6.57 | 6.14 | 9.2 | 3.82 |
| 6.24 | 3.19 | 4.38 | 4.54 | 7.44 | 9.15 | 5.53 |
| 5.41 | 3.19 | 6.82 | 5.39 | 6.97 | 7.29 | 6.17 |
| 4.32 | 0.92 | 4.26 | 3.09 | 4.88 | 6.29 | 6.21 |
| 4.37 | 3.21 | 6.35 | 5.15 | 7.31 | 7.43 | 5.85 |
| 7.13 | 3.76 | 4.65 | 6 | 5.96 | 6.24 | 3.96 |
| 6.34 | 3.49 | 3.96 | 3.71 | 9.1 | 7.34 | 4.13 |
| 2.52 | 1.07 | 6.45 | 4.26 | 7.88 | 7.33 | 3.63 |
| 5.69 | 0.98 | 4.15 | 5.52 | 4.88 | 8.87 | 3.54 |
| 5.24 | 1.13 | 5.65 | 3.13 | 7.79 | 10.18 | 3.54 |
| 4.62 | 1.35 | 2.94 | 5.33 | 8.51 | 7.78 | 4.4 |
| 2.68 | 3.74 | 5.57 | 2.18 | 8.51 | 4.38 | 3.54 |
| 3.53 | 3.12 | 5.77 | 3.15 | 5.19 | 4.32 | 3.85 |
| 5.29 | 1.28 | 4.05 | 7.07 | 4.92 | 8.56 | 3.67 |
| 6.09 | 1.08 | 5.78 | 5.58 | 6.13 | 4.26 | 6.48 |
| 3.83 | 2.89 | 4.28 | 6.29 | 5.47 | 6.27 | 5.89 |
| 2.89 | 0.61 | 3.33 | 4.05 | 7.02 | 5.74 | 3.54 |
| 5.67 | 1.72 | 5.77 | 3.84 | 4.88 | 7.1 | 6.89 |
| 2.97 | 1.12 | 5.7 | 6 | 7.51 | 5.53 | 5.61 |
| 5.96 | 3.57 | 5.68 | 3.33 | 4.88 | 6.43 | 3.84 |
| 3.22 | 0.87 | 4.38 | 5.1 | 6.01 | 8.68 | 3.97 |
| 1.74 | 3.84 | 5.15 | 6.25 | 7.12 | 4.73 | 3.54 |
| 7.85 | 3.32 | 4.39 | 2.91 | 7.11 | 7.47 | 3.54 |
| 4.62 | 0.34 | 2.36 | 2.18 | 4.88 | 3.71 | 3.54 |
| 3.6 | 3.84 | 6.83 | 7.07 | 10.13 | 10.96 | 7.18 |
