# Supplementary material for: The effects of L-carnitine and fructose in improved Ham’s F10 on sperm culture in idiopathic severe asthenospermia within 24h
Source: PLoS One. 2025 Feb 10;20(2):e0306235. doi: 10.1371/journal.pone.0306235 (PMC11809793; doi:10.1371/journal.pone.0306235)
Supplement: S3 File — (DOCX) [file pone.0306235.s003.docx]

c1: Percentage of forward motility of sperm (%)

| basic data 3+A2:D55 | NS3-30min | HF3-30min | m-HF-30min |
| --- | --- | --- | --- |
| 7.12 | 5.67 | 9.58 | 9.15 |
| 6.99 | 6.4 | 8.03 | 11.74 |
| 6.31 | 7.05 | 7.41 | 12.95 |
| 6.66 | 7 | 8.92 | 10.83 |
| 8.51 | 6.5 | 8 | 11.83 |
| 6.86 | 6.95 | 11 | 11.89 |
| 6.78 | 8.44 | 12.27 | 8.94 |
| 7.52 | 7.39 | 9.79 | 8.11 |
| 4.47 | 6.45 | 7.41 | 12.39 |
| 8.4 | 6.92 | 10.97 | 10.46 |
| 4.86 | 6.28 | 11.65 | 9.98 |
| 6.05 | 5.97 | 11.89 | 13.37 |
| 5.28 | 6.32 | 12.81 | 14.26 |
| 5.35 | 7.13 | 7.77 | 14.71 |
| 7.19 | 8.03 | 11.89 | 11.81 |
| 7.4 | 6.84 | 9.46 | 13.23 |
| 6.78 | 7.84 | 13.12 | 11.19 |
| 8.04 | 4.68 | 10.72 | 8.72 |
| 6.39 | 7.87 | 9.28 | 12.17 |
| 6.16 | 7.82 | 8.69 | 13.63 |
| 7.61 | 7.59 | 11.05 | 10.02 |
| 7.14 | 8.65 | 7.41 | 12.69 |
| 7.77 | 5.26 | 12.44 | 11.23 |
| 7.31 | 8.58 | 9.05 | 13.3 |
| 7.91 | 6.51 | 11.04 | 10.84 |
| 5.91 | 7.82 | 9.06 | 9.81 |
| 7.53 | 5.28 | 12.23 | 7.96 |
| 5.62 | 5.32 | 8.96 | 14.21 |
| 7.15 | 6.22 | 12.82 | 8.15 |
| 4.68 | 7.08 | 11.08 | 7.9 |
| 8.87 | 4.72 | 11.63 | 14.69 |
| 7.33 | 6.65 | 8.37 | 15.2 |
| 7.21 | 6.12 | 8.54 | 14.53 |
| 5.93 | 8.5 | 7.41 | 11.16 |
| 7.69 | 5.9 | 14.06 | 12.97 |
| 8.74 | 8.07 | 7.43 | 10.63 |
| 6.75 | 6.24 | 11.74 | 12.68 |
| 5.4 | 7.02 | 7.41 | 14.04 |
| 5.89 | 6.2 | 11.79 | 14.41 |
| 7.53 | 5.64 | 11.55 | 8.16 |
| 6.5 | 5.32 | 13.18 | 11.45 |
| 8.2 | 5.2 | 13.73 | 13.6 |
| 7.16 | 8.68 | 9.55 | 12.03 |
| 6.93 | 8.87 | 8.56 | 10.52 |
| 5.6 | 4.49 | 7.99 | 12.7 |
| 7.81 | 4.97 | 12.65 | 11.82 |
| 5.31 | 5.32 | 9.75 | 13.98 |
| 7.86 | 7.52 | 11.79 | 12.75 |
| 7.08 | 6.47 | 8.02 | 10.41 |
| 7.88 | 5.59 | 8.76 | 11.93 |
| 6.56 | 7.32 | 8.26 | 13.39 |
| 7 | 6.84 | 13.25 | 10.55 |
| 5.89 | 7.41 | 9.61 | 8.46 |
| 7.42 | 7.66 | 8.7 | 10.31 |
| 7.4 | 9.04 | 8.59 | 11.84 |
| 5.96 | 8.06 | 11.11 | 14.62 |
| 8.19 | 5.6 | 13.37 | 12.7 |
| 7.5 | 7.45 | 15.02 | 9.34 |
| 6.67 | 4.49 | 7.41 | 6.37 |

c2: Percentage of non-forward motile sperm (%)

| basic data 3+F2:H52 | NS3-30min | HF3-30min | m-HF-30min |
| --- | --- | --- | --- |
| 6.15 | 4.66 | 8.01 | 12.68 |
| 6.73 | 5.64 | 11.13 | 7.86 |
| 4.96 | 4.18 | 8 | 8.88 |
| 5.54 | 3.9 | 12.7 | 7.25 |
| 6 | 6.45 | 8.37 | 9.43 |
| 5.65 | 5.55 | 11.47 | 9.81 |
| 3.34 | 6.84 | 8.69 | 13.37 |
| 2.74 | 3.68 | 11.7 | 9.81 |
| 6.65 | 5.48 | 8.53 | 12.69 |
| 5.07 | 4 | 7.18 | 11.92 |
| 4.51 | 3.44 | 7.89 | 8.26 |
| 5.61 | 6.66 | 8.57 | 7.25 |
| 4.5 | 5.97 | 11.21 | 13.68 |
| 5.26 | 6.48 | 7.26 | 8.96 |
| 6 | 4.91 | 7.15 | 11.19 |
| 5.45 | 4.63 | 11.79 | 9.24 |
| 3.87 | 3.65 | 11.36 | 11.65 |
| 3.98 | 6.08 | 11.04 | 8.55 |
| 6.49 | 6.79 | 7.15 | 9.63 |
| 4.06 | 3.34 | 12.72 | 12.95 |
| 6.57 | 5.8 | 8.75 | 14.59 |
| 4.2 | 3.31 | 7.15 | 9.72 |
| 4.56 | 6.62 | 11.3 | 12.17 |
| 4.14 | 5.5 | 10.53 | 14.48 |
| 4.61 | 4.38 | 8.01 | 12.29 |
| 4.73 | 5.73 | 7.36 | 9.71 |
| 4.35 | 6.66 | 7.37 | 8.35 |
| 4.9 | 5.6 | 7.15 | 11.72 |
| 6.31 | 3.53 | 8.32 | 12.78 |
| 5.72 | 3.74 | 7.15 | 9.03 |
| 2.75 | 6.24 | 8.23 | 7.56 |
| 6.23 | 6.3 | 7.15 | 12.26 |
| 4.09 | 5.81 | 10.02 | 11.26 |
| 5.8 | 6.87 | 11.04 | 9.25 |
| 6.36 | 4.78 | 8.9 | 9.19 |
| 5.22 | 4.83 | 8.35 | 7.25 |
| 5.14 | 5.55 | 10.23 | 12.23 |
| 5.3 | 4.27 | 8.3 | 13.03 |
| 4.87 | 5.66 | 8.57 | 8.61 |
| 5.42 | 5.83 | 12.29 | 11.7 |
| 4.91 | 6.54 | 10.2 | 13.03 |
| 5.53 | 4.49 | 12.36 | 8.23 |
| 5.55 | 3.73 | 10.06 | 9.77 |
| 5.13 | 3.98 | 10.55 | 11.13 |
| 3.75 | 3.99 | 8.57 | 9.52 |
| 5.79 | 4.26 | 11.29 | 9.74 |
| 6.31 | 4.74 | 7.33 | 11.46 |
| 4.47 | 5.51 | 7.81 | 9.29 |
| 5.34 | 6.54 | 8.1 | 8.93 |
| 4.13 | 6.41 | 11.45 | 11.49 |
| 3.54 | 5.4 | 8.2 | 9.67 |
| 4.97 | 4.92 | 10.9 | 12.97 |
| 4.41 | 6.87 | 10.29 | 12.95 |
| 6.75 | 5.46 | 8.55 | 9.77 |
| 5.59 | 5.41 | 10.67 | 13.58 |
| 6.19 | 3.47 | 7.87 | 7.55 |
| 2.86 | 5.86 | 11.28 | 7.49 |
| 4.21 | 5.21 | 10.71 | 11.45 |
| 4.9 | 3.31 | 7.15 | 7.25 |
| 4.53 | 6.87 | 13.46 | 14.59 |

c3: Deformity rate (%)

| basic data 3+K2:M52 | NS3-30min | HF3-30min | m-HF-30min |
| --- | --- | --- | --- |
| 87.75 | 87.67 | 87.2 | 83.94 |
| 86.43 | 88.22 | 86.92 | 86.68 |
| 86.72 | 87.76 | 87.82 | 89.15 |
| 88.77 | 88.36 | 89.37 | 88.17 |
| 87.36 | 84.95 | 87.07 | 92.32 |
| 89.24 | 90.07 | 86.92 | 85.13 |
| 87.9 | 88.47 | 88.42 | 84.96 |
| 87.77 | 88.22 | 88.48 | 87.83 |
| 88.4 | 86.9 | 86.88 | 87.83 |
| 86.92 | 85.35 | 89.85 | 90.29 |
| 87.79 | 88.02 | 87 | 88.09 |
| 89.81 | 89.18 | 87.5 | 88.05 |
| 88.22 | 85.54 | 86.74 | 90.93 |
| 89.38 | 88.16 | 89.68 | 89.58 |
| 89.27 | 87.44 | 89.95 | 89.17 |
| 88.82 | 87.11 | 87.8 | 87.83 |
| 87.55 | 89.15 | 89.06 | 86.56 |
| 89.69 | 87.99 | 88.47 | 84.47 |
| 85.49 | 87.73 | 88.49 | 90.52 |
| 89.19 | 85.98 | 86.86 | 87.83 |
| 85.12 | 86.81 | 86.88 | 88.45 |
| 88.54 | 88.24 | 87 | 87.83 |
| 87.82 | 87.65 | 87.05 | 87.55 |
| 90 | 87.69 | 87.12 | 87.57 |
| 90.95 | 87.56 | 86.21 | 88.3 |
| 86.11 | 89.76 | 87.88 | 86.83 |
| 88.75 | 88.72 | 89.63 | 90.33 |
| 87.69 | 88.02 | 89.14 | 85.32 |
| 90.33 | 88.6 | 85.99 | 91.24 |
| 89.55 | 90.27 | 87.18 | 86.44 |
| 89.08 | 86.28 | 87.84 | 87.27 |
| 87.81 | 88.85 | 87.33 | 86.37 |
| 86.15 | 89.62 | 88.39 | 87.4 |
| 88.84 | 87.74 | 87.29 | 86.67 |
| 89.09 | 89 | 87.7 | 87.57 |
| 89.65 | 89.57 | 88.42 | 89.78 |
| 86.14 | 87.99 | 86.86 | 87.83 |
| 88.9 | 85.19 | 88.27 | 86.94 |
| 88.26 | 89.97 | 87.31 | 86.19 |
| 86.96 | 88.55 | 86.71 | 86.27 |
| 89.16 | 89.36 | 88.33 | 86.87 |
| 88.91 | 88.2 | 89.1 | 92.89 |
| 87.99 | 87.99 | 88.9 | 87.49 |
| 91.22 | 86.22 | 88.65 | 87.05 |
| 87.66 | 86.52 | 88.46 | 87.69 |
| 87.31 | 87.2 | 88.67 | 91.25 |
| 87.34 | 88.21 | 88.01 | 87.81 |
| 86.12 | 87.99 | 88.87 | 87.83 |
| 88.75 | 88.09 | 88.51 | 86.41 |
| 85.59 | 86.2 | 87.5 | 86.63 |
| 89.1 | 89.07 | 88.87 | 93.71 |
| 85.17 | 90.23 | 87.78 | 87.83 |
| 88.27 | 90.83 | 89.46 | 85.35 |
| 89.57 | 89.22 | 88.64 | 88.68 |
| 86.94 | 87.34 | 87.78 | 90.15 |
| 89.47 | 89.32 | 89.91 | 84.72 |
| 86.28 | 88.94 | 87.78 | 84.79 |
| 86.8 | 85.23 | 85.96 | 86.91 |
| 89.23 | 88.29 | 88.69 | 86.72 |
| 87.8 | 86.3 | 89.75 | 87.83 |

c4: Deformity rate (%)

| basic data 3+P2:S54 | NS3-30min | HF3-30min | m-HF-30min |
| --- | --- | --- | --- |
| 67.62 | 74.19 | 70.01 | 64.51 |
| 79.29 | 77.27 | 68.58 | 64.21 |
| 68.98 | 68.93 | 59.87 | 65.33 |
| 71.16 | 64.37 | 67.24 | 74.62 |
| 69.62 | 65.93 | 70.01 | 71 |
| 55.25 | 75.94 | 68.17 | 77.11 |
| 74.49 | 75.92 | 73.01 | 74.79 |
| 74.95 | 62.11 | 77.86 | 67.47 |
| 75.37 | 65.32 | 65.73 | 72.11 |
| 71.58 | 73.57 | 77.38 | 69.67 |
| 63.77 | 67.08 | 65.72 | 67.33 |
| 62.33 | 68.93 | 68.73 | 62.82 |
| 69.44 | 68.93 | 75.5 | 66.74 |
| 72.14 | 61.98 | 78.89 | 73.38 |
| 70.29 | 75.36 | 61.01 | 65.61 |
| 62.1 | 63.54 | 73.1 | 72.55 |
| 71.1 | 72.61 | 73.17 | 66.18 |
| 82.05 | 66.56 | 75.98 | 67.51 |
| 68.97 | 68.93 | 75.37 | 73.89 |
| 69.58 | 62.49 | 63.33 | 69.99 |
| 63.13 | 68.93 | 70.54 | 74.76 |
| 62.62 | 65.66 | 73.54 | 75.93 |
| 69.13 | 76.44 | 71.4 | 67.29 |
| 71.33 | 71.64 | 60.36 | 75.29 |
| 72.19 | 68.14 | 67.43 | 67.61 |
| 64.38 | 67.85 | 70.01 | 64.83 |
| 71.31 | 68.3 | 68.79 | 66.56 |
| 63.33 | 75.79 | 71.77 | 67.51 |
| 66 | 63.04 | 66.8 | 61.78 |
| 68.7 | 77.5 | 68.68 | 77.47 |
| 64.78 | 65.15 | 70.01 | 65.13 |
| 58.74 | 61.85 | 70.01 | 73.94 |
| 67.28 | 66.68 | 70.01 | 61.6 |
| 69.63 | 68.93 | 67.4 | 72.12 |
| 68.64 | 65.17 | 61.62 | 74.75 |
| 66.62 | 67.76 | 66.03 | 72.99 |
| 72.05 | 68.93 | 69.67 | 70.81 |
| 72.96 | 65.41 | 69.51 | 72.79 |
| 66.59 | 72.17 | 66.3 | 67.95 |
| 69.43 | 67.6 | 77.46 | 76.83 |
| 77.31 | 61.8 | 72.52 | 65.29 |
| 66.58 | 59.04 | 66.92 | 62.73 |
| 58.99 | 64.34 | 66.88 | 60.31 |
| 60.33 | 70.46 | 69.66 | 62.36 |
| 76.88 | 70.97 | 75.05 | 69.82 |
| 69.62 | 66.67 | 69.85 | 65.9 |
| 73.02 | 67.27 | 72.03 | 76.37 |
| 70.31 | 78.69 | 70.6 | 64.22 |
| 69.22 | 66.06 | 73.29 | 71.87 |
| 61.37 | 69.12 | 77.21 | 61.04 |
| 74.51 | 82.37 | 70.39 | 74.79 |
| 66.16 | 61.88 | 69.03 | 65.76 |
| 70.52 | 69.7 | 69.92 | 60.31 |
| 72.13 | 63.75 | 70.01 | 67.02 |
| 69.82 | 73.74 | 70.01 | 74.96 |
| 65.81 | 71.01 | 62.12 | 73.46 |
| 76.54 | 71.93 | 74.99 | 70.69 |
| 71.91 | 67.97 | 76.72 | 74.07 |
| 77.17 | 54.35 | 56.76 | 60.31 |
| 74.59 | 91.49 | 80.34 | 77.47 |

c5: Sperm DNA fragmentation rate (%)

| basic data 3 | NS3-30min | HF3-30min | m-HF-30min |
| --- | --- | --- | --- |
| 16.9 | 19.04 | 19.39 | 17.62 |
| 16.08 | 15.59 | 15.29 | 17.01 |
| 16.5 | 19.69 | 16.06 | 16.05 |
| 16.91 | 16.66 | 19.22 | 15.25 |
| 15.63 | 15.71 | 15.93 | 18.09 |
| 14.75 | 16.05 | 16.23 | 16.8 |
| 15.41 | 16.9 | 17.85 | 18.53 |
| 16.14 | 19.46 | 14.94 | 17.48 |
| 20.1 | 18.23 | 17.82 | 18.51 |
| 16.69 | 16.89 | 15.26 | 16.35 |
| 19.09 | 18.38 | 17.8 | 15.73 |
| 16.82 | 18.04 | 17.33 | 17.73 |
| 14.89 | 18.53 | 19.39 | 17.41 |
| 17.89 | 19.31 | 17.26 | 17.26 |
| 15.86 | 19.33 | 18.31 | 15.91 |
| 18.67 | 19.69 | 15.12 | 15.91 |
| 15.99 | 15.82 | 18.42 | 16.55 |
| 17.2 | 19.69 | 15.37 | 18.76 |
| 17.59 | 18.55 | 17.84 | 18.07 |
| 16.99 | 16.68 | 17.67 | 16.53 |
| 18.75 | 17.41 | 16.63 | 17.81 |
| 15.21 | 19.24 | 17.75 | 18.78 |
| 18.14 | 19.49 | 16.08 | 15.8 |
| 17.93 | 18.54 | 17.52 | 16.72 |
| 15.07 | 16.94 | 16.35 | 18.59 |
| 16.97 | 19.63 | 14.44 | 18.69 |
| 15.24 | 19.69 | 17.58 | 17.28 |
| 17.3 | 15.2 | 16.65 | 18.47 |
| 19.18 | 18.91 | 18.83 | 15.8 |
| 15.83 | 16.63 | 17.81 | 17.6 |
| 17.03 | 16.83 | 14.44 | 17.19 |
| 16.94 | 18.13 | 16.03 | 16.64 |
| 16.13 | 16.79 | 16.54 | 16.34 |
| 17.47 | 19.69 | 17.89 | 16.83 |
| 18.42 | 17.2 | 15.76 | 18.18 |
| 15.31 | 18.61 | 17.91 | 17.54 |
| 16.69 | 18.73 | 19.1 | 17.24 |
| 18.43 | 17.11 | 17.68 | 17.4 |
| 15.62 | 15.33 | 14.8 | 15.17 |
| 18.15 | 18.77 | 16.72 | 17.61 |
| 20.47 | 15.47 | 18.09 | 18.39 |
| 18.53 | 16.74 | 18.66 | 17.11 |
| 17.62 | 18.6 | 16.46 | 17.08 |
| 17.73 | 19.69 | 16.06 | 17.21 |
| 15.62 | 17.6 | 15.91 | 15.92 |
| 17.02 | 15.64 | 18.95 | 18.41 |
| 16.36 | 15.88 | 19.18 | 18.78 |
| 17.86 | 18.69 | 15.37 | 15.61 |
| 15.65 | 16.37 | 18.86 | 15.36 |
| 15.75 | 19.1 | 17.76 | 18.65 |
| 17.37 | 18.23 | 16.71 | 16.77 |
| 20.28 | 18.11 | 18.14 | 17.9 |
| 18.15 | 15.17 | 17.73 | 18.24 |
| 15.26 | 19.4 | 16.36 | 17.97 |
| 19.7 | 17.16 | 19.15 | 17.45 |
| 17.81 | 19.46 | 15.77 | 18.1 |
| 19.58 | 19.37 | 14.6 | 17.06 |
| 15.46 | 16.37 | 16.31 | 16.7 |
| 16.88 | 14.44 | 14.44 | 14.77 |
| 16.1 | 19.7 | 19.77 | 18.79 |
