# Supplementary material for: The effects of L-carnitine and fructose in improved Ham’s F10 on sperm culture in idiopathic severe asthenospermia within 24h
Source: PLoS One. 2025 Feb 10;20(2):e0306235. doi: 10.1371/journal.pone.0306235 (PMC11809793; doi:10.1371/journal.pone.0306235)
Supplement: S4 File — (DOCX) [file pone.0306235.s004.docx]

d1: Percentage of forward motility of sperm (%)

| basic data 4 | NS3-1h | HF3-1h | m-HF -1h |
| --- | --- | --- | --- |
| 7.12 | 4.89 | 12.22 |  |
| 6.99 | 8.55 | 10.46 | 9.92 |
| 6.31 | 7.56 | 11.53 | 13.8 |
| 6.66 | 5.19 | 10.08 | 9.93 |
| 8.51 | 7.39 | 10.45 | 13.65 |
| 6.86 | 4.89 | 9.46 | 8.04 |
| 6.78 | 8.65 | 10.01 | 8.53 |
| 7.52 | 7.52 | 9.42 | 9.92 |
| 4.47 | 7.2 | 9.52 | 10.51 |
| 8.4 | 5.98 | 11.14 | 10.51 |
| 4.86 | 5.52 | 9.76 | 11.77 |
| 6.05 | 8.68 | 9.67 | 8.92 |
| 5.28 | 8.23 | 12.08 | 10.52 |
| 5.35 | 7.63 | 9.92 | 8.61 |
| 7.19 | 5.98 | 12.1 | 7.79 |
| 7.4 | 8.2 | 11.45 | 11.76 |
| 6.78 | 5.59 | 12.54 | 12.59 |
| 8.04 | 8.25 | 8.5 | 15.26 |
| 6.39 | 7.67 | 12.36 | 8.72 |
| 6.16 | 5.1 | 10 | 12.91 |
| 7.61 | 6.39 | 10.02 | 9.6 |
| 7.14 | 7.71 | 11.05 | 12.02 |
| 7.77 | 6.33 | 9.86 | 13.37 |
| 7.31 | 8.27 | 12.5 | 13.22 |
| 7.91 | 6.45 | 9.84 | 9.52 |
| 5.91 | 7.23 | 10.22 | 10.08 |
| 7.53 | 7.22 | 12.54 | 9.82 |
| 5.62 | 8.19 | 12.54 | 13.23 |
| 7.15 | 7.93 | 11.94 | 15.89 |
| 4.68 | 5.44 | 11.75 | 12.73 |
| 8.87 | 6.39 | 11.05 | 12.25 |
| 7.33 | 5.98 | 12.05 | 10.63 |
| 7.21 | 5.42 | 10.92 | 11.82 |
| 5.93 | 7.09 | 9.62 | 10.23 |
| 7.69 | 7.33 | 11.77 | 10.24 |
| 8.74 | 7.34 | 11.43 | 8.03 |
| 6.75 | 4.89 | 10.37 | 12.46 |
| 5.4 | 8.38 | 10.03 | 9.54 |
| 5.89 | 7.96 | 9.59 | 13.58 |
| 7.53 | 7.74 | 10.26 | 13.67 |
| 6.5 | 6.01 | 9.78 | 9.15 |
| 8.2 | 8.51 | 9.65 | 14.02 |
| 7.16 | 5.48 | 10.52 | 13.08 |
| 6.93 | 5.98 | 9.44 | 9.43 |
| 5.6 | 6.83 | 10.87 | 12.79 |
| 7.81 | 5.64 | 9.53 | 14.76 |
| 5.31 | 6.02 | 11.29 | 13.11 |
| 7.86 | 5.4 | 11.87 | 10.6 |
| 7.08 | 8.39 | 10.97 | 8.86 |
| 7.88 | 6.57 | 9.18 | 12.66 |
| 6.56 | 6.21 | 10.93 | 10.04 |
| 7 | 5.76 | 9.68 | 7.24 |
| 5.89 | 4.89 | 12.25 | 8.12 |
| 7.42 | 7.6 | 11.99 | 12.47 |
| 7.4 | 7.67 | 11.33 | 8.94 |
| 5.96 | 6.42 | 11.25 | 12.63 |
| 8.19 | 7.63 | 10.3 | 12.75 |
| 7.5 | 7.11 | 11.28 | 12.89 |
| 6.67 | 4.89 | 8.04 | 7.12 |
| 5.3 | 8.73 | 12.54 | 16.09 |

d2: Percentage of non-forward motile sperm (%)

| basic data 4+F2:I52 | NS3-1h | HF3-1h | m-HF -1h |
| --- | --- | --- | --- |
| 6.15 | 3.46 | 11.38 | 10.67 |
| 6.73 | 4.62 | 9.12 | 11.35 |
| 4.96 | 6.24 | 9.15 | 13.45 |
| 5.54 | 6.36 | 8.39 | 11.79 |
| 6 | 6.74 | 11.92 | 13.24 |
| 5.65 | 7.02 | 8.92 | 9.4 |
| 3.34 | 4.06 | 10.94 | 11.53 |
| 2.74 | 4.89 | 9.34 | 11.16 |
| 6.65 | 4.46 | 9.35 | 9.9 |
| 5.07 | 4.06 | 10.63 | 10.86 |
| 4.51 | 4.22 | 9.52 | 11.24 |
| 5.61 | 3.78 | 9.19 | 9.51 |
| 4.5 | 6.19 | 9.6 | 7.64 |
| 5.26 | 4.48 | 12.45 | 11.13 |
| 6 | 5.73 | 8.9 | 12.18 |
| 5.45 | 6.98 | 11.46 | 12.11 |
| 3.87 | 6.68 | 9.73 | 9.85 |
| 3.98 | 3.01 | 12.38 | 12.22 |
| 6.49 | 3.04 | 10.97 | 12.43 |
| 4.06 | 3.43 | 10.49 | 13.01 |
| 6.57 | 4.2 | 12.67 | 8.65 |
| 4.2 | 3.63 | 8.85 | 12.13 |
| 4.56 | 5.9 | 11.72 | 11.18 |
| 4.14 | 7.18 | 9.41 | 8.51 |
| 4.61 | 6.57 | 10.93 | 8 |
| 4.73 | 5.59 | 10.68 | 9.05 |
| 4.35 | 4.51 | 10.44 | 11.94 |
| 4.9 | 5.8 | 11.72 | 10.74 |
| 6.31 | 4.75 | 9.37 | 9.77 |
| 5.72 | 4.56 | 11.11 | 9.11 |
| 2.75 | 2.86 | 9.78 | 8.63 |
| 6.23 | 6.21 | 8.84 | 10.63 |
| 4.09 | 6.66 | 9.01 | 9.67 |
| 5.8 | 4.02 | 9.26 | 10.95 |
| 6.36 | 6.79 | 9.65 | 7.8 |
| 5.22 | 5.76 | 8.89 | 11.96 |
| 5.14 | 3.02 | 10.93 | 9.46 |
| 5.3 | 6.85 | 11.52 | 9.44 |
| 4.87 | 5.88 | 9.1 | 9.11 |
| 5.42 | 6.12 | 9.97 | 11.64 |
| 4.91 | 4.06 | 11.54 | 7.39 |
| 5.53 | 4.43 | 9.92 | 10.76 |
| 5.55 | 6.57 | 10.66 | 10.21 |
| 5.13 | 5.8 | 8.89 | 12.64 |
| 3.75 | 4.6 | 10.89 | 9.74 |
| 5.79 | 7 | 8.99 | 13.45 |
| 6.31 | 6.33 | 9.55 | 9.5 |
| 4.47 | 3.16 | 11.5 | 8.17 |
| 5.34 | 4.46 | 8.08 | 8.79 |
| 4.13 | 6.13 | 11.86 | 8.99 |
| 3.54 | 6.67 | 8.88 | 7.36 |
| 4.97 | 6.06 | 10.72 | 12.46 |
| 4.41 | 6.66 | 12.54 | 8.8 |
| 6.75 | 6.32 | 10.69 | 10.67 |
| 5.59 | 5.73 | 11.17 | 8 |
| 6.19 | 5.69 | 10.82 | 13.27 |
| 2.86 | 3.8 | 8.72 | 8.74 |
| 4.21 | 3.5 | 8.08 | 12.18 |
| 4.9 | 2.86 | 8.08 | 7.29 |
| 4.53 | 7.37 | 13.63 | 13.45 |

d3: Deformity rate (%)

| basic data 4+K2:M54 | NS3-1h | HF3-1h | m-HF -1h |
| --- | --- | --- | --- |
| 89.22 | 86.14 | 87.84 | 85.68 |
| 88.31 | 87.75 | 86.28 | 85.19 |
| 86.93 | 88.19 | 87.86 | 87.64 |
| 88.11 | 86.09 | 86.71 | 86.41 |
| 90.7 | 89.04 | 88.26 | 85.88 |
| 89.06 | 85.38 | 87.99 | 88.65 |
| 89.56 | 87.82 | 86.68 | 88.5 |
| 88.39 | 85.38 | 86.48 | 84.94 |
| 87.04 | 85.57 | 87.83 | 86.19 |
| 88.74 | 86.34 | 87.51 | 87.14 |
| 88.63 | 86.39 | 85.74 | 86.82 |
| 89.94 | 86.2 | 88.03 | 88.62 |
| 85.57 | 85.78 | 87.6 | 88.14 |
| 88.6 | 88.18 | 86.56 | 85.14 |
| 87.95 | 89.27 | 84.68 | 88.09 |
| 89.59 | 88.55 | 88.87 | 85.73 |
| 87.1 | 85.73 | 86.73 | 86.82 |
| 89.71 | 89.64 | 85.88 | 86.24 |
| 87.72 | 86.43 | 88.87 | 86.04 |
| 87.98 | 85.38 | 88.86 | 86.51 |
| 86.72 | 86.27 | 86.38 | 86.52 |
| 89.44 | 86.5 | 88.65 | 86.54 |
| 90.56 | 85.7 | 86.46 | 88.2 |
| 87.14 | 88.36 | 86 | 85.82 |
| 86.03 | 88.41 | 87.48 | 87.88 |
| 86.06 | 85.7 | 88.27 | 87.38 |
| 87.04 | 86.74 | 87.91 | 86.12 |
| 88.11 | 87.95 | 88.12 | 86.88 |
| 87.74 | 88.3 | 88.87 | 88.26 |
| 91.24 | 85.38 | 88.7 | 87.52 |
| 87.81 | 85.38 | 84.76 | 88.15 |
| 86.52 | 88.12 | 85.83 | 87.01 |
| 90.85 | 88.04 | 86.54 | 86 |
| 88.66 | 86.44 | 86.32 | 85.67 |
| 88.55 | 88.04 | 88.87 | 88.57 |
| 88.11 | 88.22 | 87.75 | 87.87 |
| 88.1 | 87.81 | 84.79 | 87.73 |
| 88.72 | 88.36 | 88.52 | 88.62 |
| 86.92 | 87.83 | 85.94 | 86.68 |
| 87.61 | 88.14 | 85.87 | 88.04 |
| 88.39 | 89.23 | 88.87 | 85.96 |
| 87.92 | 88.36 | 86.51 | 87.91 |
| 86.61 | 86 | 85.37 | 86.35 |
| 88.07 | 86.49 | 84.77 | 88.54 |
| 91.26 | 85.38 | 88.77 | 86.79 |
| 87.09 | 88.45 | 85.38 | 86.62 |
| 85.71 | 85.57 | 85.64 | 87.18 |
| 87.64 | 88.72 | 88.15 | 85.93 |
| 87.08 | 87.92 | 86.23 | 87.26 |
| 87.3 | 86.02 | 87.65 | 85.66 |
| 84.83 | 88.17 | 84.93 | 88.73 |
| 88.94 | 86.56 | 85.2 | 88.67 |
| 86.03 | 87.77 | 86.78 | 87.45 |
| 86.39 | 85.38 | 85.91 | 87.65 |
| 89.24 | 86.08 | 88.87 | 85.18 |
| 90.1 | 89.3 | 86.53 | 86.77 |
| 88.11 | 87.64 | 88.44 | 86.5 |
| 89.32 | 85.56 | 87.81 | 87.84 |
| 88.32 | 85.38 | 84.13 | 84.69 |
| 87.76 | 89.77 | 88.88 | 88.8 |

d4: Survival rate (%)

| basic data 4 | NS3-1h | HF3-1h | m-HF -1h |
| --- | --- | --- | --- |
| 67.62 | 75.47 | 62.6 | 64.95 |
| 79.29 | 65.27 | 67.68 | 72.9 |
| 68.98 | 71.94 | 75.48 | 74.68 |
| 71.16 | 66.45 | 67.12 | 68.27 |
| 69.62 | 63.5 | 68.01 | 69.1 |
| 55.25 | 72.04 | 68.6 | 72.9 |
| 74.49 | 72.39 | 72.63 | 75.25 |
| 74.95 | 75.73 | 75.75 | 72.31 |
| 75.37 | 66.23 | 69.39 | 61.91 |
| 71.58 | 65.11 | 69.24 | 73.18 |
| 63.77 | 63.64 | 66.6 | 68.16 |
| 62.33 | 79.9 | 66.62 | 73.09 |
| 69.44 | 78.2 | 64.61 | 76.96 |
| 72.14 | 66.05 | 64.99 | 75.48 |
| 70.29 | 79.44 | 73.89 | 76.47 |
| 62.1 | 72.3 | 75.81 | 72.51 |
| 71.1 | 73.15 | 64.88 | 74.51 |
| 82.05 | 64.57 | 75.15 | 69.09 |
| 68.97 | 66.6 | 64.69 | 67.98 |
| 69.58 | 72.08 | 63.71 | 68.98 |
| 63.13 | 76.95 | 63.6 | 69.99 |
| 62.62 | 69.01 | 67.3 | 75.39 |
| 69.13 | 67.01 | 76.96 | 68.92 |
| 71.33 | 68.08 | 73.57 | 66.89 |
| 72.19 | 75.61 | 67.74 | 66.21 |
| 64.38 | 65.8 | 64.91 | 67.43 |
| 71.31 | 64.97 | 74.02 | 66.9 |
| 63.33 | 66.86 | 78.38 | 58.54 |
| 66 | 77.96 | 78.9 | 66.69 |
| 68.7 | 67.34 | 64.65 | 75.28 |
| 64.78 | 75.99 | 78.53 | 76.3 |
| 58.74 | 72.94 | 76.96 | 74.81 |
| 67.28 | 74.61 | 68.85 | 73.94 |
| 69.63 | 62.79 | 79.12 | 62.82 |
| 68.64 | 73.06 | 71.48 | 77.44 |
| 66.62 | 74.53 | 75.62 | 73.24 |
| 72.05 | 72.86 | 72.74 | 65.43 |
| 72.96 | 71.61 | 68.23 | 64.01 |
| 66.59 | 68.4 | 72.52 | 66.36 |
| 69.43 | 73.05 | 74.3 | 74.45 |
| 77.31 | 72.73 | 68.27 | 68.76 |
| 66.58 | 67.67 | 72.83 | 68.64 |
| 58.99 | 68.85 | 68.77 | 69.36 |
| 60.33 | 76.47 | 66.95 | 66.98 |
| 76.88 | 78.6 | 74.41 | 71.37 |
| 69.62 | 69.45 | 68.33 | 70.96 |
| 73.02 | 73.19 | 66.32 | 73.55 |
| 70.31 | 71.87 | 67.17 | 73.53 |
| 69.22 | 67.58 | 75.07 | 65.97 |
| 61.37 | 73.3 | 67.24 | 67.87 |
| 74.51 | 66.85 | 68.55 | 72.17 |
| 66.16 | 79.19 | 64.02 | 76.21 |
| 70.52 | 65.15 | 72.85 | 66.73 |
| 72.13 | 61.7 | 65.25 | 70.02 |
| 69.82 | 72.39 | 73.42 | 69.53 |
| 65.81 | 64.36 | 62.23 | 69.84 |
| 76.54 | 68.11 | 78.31 | 68.32 |
| 71.91 | 64.02 | 72.93 | 76.59 |
| 77.17 | 60.62 | 61.74 | 57.18 |
| 74.59 | 79.91 | 80.8 | 77.99 |

d5: Sperm DNA fragmentation rate (%)

| basic data 4 | NS3-1h | HF3-1h | m-HF -1h |
| --- | --- | --- | --- |
| 16.9 | 17.22 | 18.57 | 15.52 |
| 16.08 | 16.94 | 18.17 | 17.35 |
| 16.5 | 17.54 | 17.57 | 15.23 |
| 16.91 | 17 | 15.2 | 18.14 |
| 15.63 | 17.61 | 15.95 | 17.33 |
| 14.75 | 19.18 | 15.53 | 17.5 |
| 15.41 | 20.02 | 17.32 | 18.53 |
| 16.14 | 17.13 | 16.62 | 18.71 |
| 20.1 | 17.37 | 14.49 | 15.62 |
| 16.69 | 17.06 | 16.03 | 15.11 |
| 19.09 | 18.72 | 15.99 | 18.44 |
| 16.82 | 18.19 | 18.75 | 14.86 |
| 14.89 | 18.13 | 14.98 | 17.48 |
| 17.89 | 17.12 | 18.9 | 14.73 |
| 15.86 | 16.59 | 16.25 | 18.45 |
| 18.67 | 19.29 | 17.72 | 16.64 |
| 15.99 | 18.84 | 17.75 | 16.78 |
| 17.2 | 18.81 | 15.35 | 17.43 |
| 17.59 | 16.06 | 18.78 | 16.33 |
| 16.99 | 16.64 | 19.32 | 17.54 |
| 18.75 | 20.72 | 16.41 | 18.36 |
| 15.21 | 16.46 | 14.78 | 15.33 |
| 18.14 | 16.94 | 17.84 | 19.25 |
| 17.93 | 16.67 | 18.1 | 17.73 |
| 15.07 | 19.12 | 17.34 | 17.45 |
| 16.97 | 16.69 | 17.33 | 19.44 |
| 15.24 | 20.64 | 17.46 | 18.28 |
| 17.3 | 15.11 | 15.82 | 18.8 |
| 19.18 | 19.55 | 19.04 | 18.6 |
| 15.83 | 20.1 | 18.32 | 16.4 |
| 17.03 | 18.21 | 17.66 | 19.81 |
| 16.94 | 16.72 | 15.47 | 16.08 |
| 16.13 | 18.48 | 15.89 | 18.08 |
| 17.47 | 16.83 | 17.7 | 16.43 |
| 18.42 | 18.94 | 14.34 | 19.81 |
| 15.31 | 15.01 | 17.43 | 16.06 |
| 16.69 | 16.98 | 18.02 | 18.07 |
| 18.43 | 19.52 | 18.92 | 18.95 |
| 15.62 | 18.39 | 16.18 | 19.05 |
| 18.15 | 18.91 | 16.4 | 17.61 |
| 20.47 | 16.51 | 16.43 | 16.44 |
| 18.53 | 20 | 18.97 | 15.01 |
| 17.62 | 16.28 | 18.55 | 15.8 |
| 17.73 | 17.96 | 16.68 | 17.96 |
| 15.62 | 19.39 | 17.83 | 16.06 |
| 17.02 | 17.45 | 18.32 | 16.3 |
| 16.36 | 20.04 | 17.28 | 16.03 |
| 17.86 | 15.81 | 14.62 | 17.8 |
| 15.65 | 16.76 | 16.49 | 16.93 |
| 15.75 | 20.41 | 18.24 | 14.81 |
| 17.37 | 20.37 | 18.98 | 18.78 |
| 20.28 | 14.98 | 17.43 | 15.17 |
| 18.15 | 19.58 | 17.96 | 16 |
| 15.26 | 20.32 | 15.88 | 16.17 |
| 19.7 | 17.18 | 18.68 | 18.6 |
| 17.81 | 20.08 | 15.18 | 16.46 |
| 19.58 | 15.94 | 18.15 | 15.92 |
| 15.46 | 17.26 | 16.27 | 15.38 |
| 16.88 | 14.47 | 13.95 | 14.34 |
| 16.1 | 21.65 | 19.53 | 20.55 |
