# Supplementary material for: The effects of L-carnitine and fructose in improved Ham’s F10 on sperm culture in idiopathic severe asthenospermia within 24h
Source: PLoS One. 2025 Feb 10;20(2):e0306235. doi: 10.1371/journal.pone.0306235 (PMC11809793; doi:10.1371/journal.pone.0306235)
Supplement: S5 File — (DOCX) [file pone.0306235.s005.docx]

e1: Percentage of forward motility of sperm (%)

| basic data 5 | NS3-12h | HF3-12h | m-HF -12h |
| --- | --- | --- | --- |
| 6.16 | 1.12 | 3.14 | 6.68 |
| 6.39 | 1.01 | 3.43 | 6.61 |
| 8.04 | 0.76 | 3.53 | 8.66 |
| 6.78 | 1.5 | 2.74 | 6.75 |
| 7.4 | 0.03 | 3.49 | 6.51 |
| 7.19 | 2.47 | 2.6 | 5.93 |
| 5.35 | 1.99 | 4.91 | 8.16 |
| 5.28 | 0.03 | 3.38 | 8.01 |
| 6.05 | 0.06 | 4.39 | 6.4 |
| 4.86 | 1.15 | 3.83 | 6.02 |
| 8.4 | 3.38 | 5.1 | 6.94 |
| 4.47 | 0.09 | 2.95 | 6.22 |
| 7.52 | 0.31 | 4.34 | 8.51 |
| 6.78 | 0.52 | 3.19 | 6.9 |
| 6.86 | 2.43 | 5.45 | 6.55 |
| 8.51 | 1.64 | 3.87 | 7.56 |
| 6.66 | 2.48 | 3.28 | 8.58 |
| 6.31 | 0.18 | 2.76 | 6.56 |
| 6.99 | 3.19 | 3.95 | 7.99 |
| 7.12 | 0.79 | 4.84 | 7.78 |
| 7.61 | 0.51 | 5.92 | 7.99 |
| 7.14 | 0.56 | 2.89 | 8.1 |
| 7.77 | 0.76 | 5.13 | 6.27 |
| 7.31 | 1.45 | 3.57 | 9.59 |
| 7.91 | 0.67 | 4.34 | 6.48 |
| 5.91 | 0.03 | 2.62 | 9.14 |
| 7.53 | 2.43 | 5.8 | 7.8 |
| 5.62 | 0.29 | 4.41 | 6.78 |
| 7.15 | 1.6 | 4.77 | 6.7 |
| 4.68 | 1.52 | 6.04 | 5.22 |
| 8.87 | 0.17 | 3.23 | 7.71 |
| 7.33 | 0.77 | 4.97 | 7.74 |
| 7.21 | 0.39 | 3.38 | 6.23 |
| 5.93 | 0.65 | 4.59 | 6.46 |
| 7.69 | 1.57 | 4.84 | 7.76 |
| 8.74 | 2.36 | 2.76 | 8.6 |
| 6.75 | 2.33 | 4.42 | 7.1 |
| 5.4 | 3.41 | 4.35 | 6.7 |
| 5.89 | 2.72 | 5.99 | 7.61 |
| 7.53 | 2.42 | 3.61 | 8.47 |
| 6.5 | 0.34 | 6.5 | 6.98 |
| 8.2 | 1.53 | 4.71 | 5.77 |
| 7.16 | 0.03 | 4.01 | 8.18 |
| 6.93 | 2.28 | 2.71 | 7.89 |
| 5.6 | 2.85 | 3.11 | 6.68 |
| 7.81 | 2.2 | 4.32 | 8.44 |
| 5.31 | 0.78 | 3.74 | 6.24 |
| 7.86 | 2.16 | 3.57 | 6.61 |
| 7.08 | 2.37 | 4.89 | 8.49 |
| 7.88 | 0.58 | 5.38 | 8.6 |
| 6.56 | 0.96 | 4.43 | 7.82 |
| 7 | 0.03 | 2.75 | 7 |
| 5.89 | 0.12 | 4.77 | 5.59 |
| 7.42 | 0.96 | 4.47 | 7.73 |
| 7.4 | 0.69 | 3.27 | 9.59 |
| 5.96 | 2.21 | 5.5 | 6.83 |
| 8.19 | 0.68 | 3.88 | 9.06 |
| 7.5 | 2.67 | 4.65 | 5.49 |
| 6.67 | 0.03 | 2.6 | 5.16 |
| 5.3 | 3.68 | 6.65 | 9.59 |

e2: Percentage of non-forward motile sperm (%)

| basic data 5 | NS3-12h | HF3-12h | m-HF -12h |
| --- | --- | --- | --- |
| 4.06 | 0.03 | 3.45 | 4.71 |
| 6.49 | 0.22 | 3.24 | 8.5 |
| 3.98 | 0.22 | 4.46 | 6.68 |
| 3.87 | 0.05 | 3.43 | 5.59 |
| 5.45 | 0.03 | 3.7 | 6.76 |
| 6 | 0.03 | 3.03 | 7.47 |
| 5.26 | 0.03 | 3.31 | 6.79 |
| 4.5 | 0.11 | 2.05 | 6.62 |
| 5.61 | 0.04 | 3.79 | 7.71 |
| 4.51 | 0.03 | 2.83 | 4.71 |
| 5.07 | 0.03 | 3.41 | 8.39 |
| 6.65 | 0.03 | 2.75 | 5.74 |
| 2.74 | 0.75 | 4.28 | 4.71 |
| 3.34 | 0.38 | 4.68 | 4.89 |
| 5.65 | 0.35 | 2.7 | 4.95 |
| 6 | 0.57 | 2.01 | 4.71 |
| 5.54 | 0.52 | 4.85 | 4.95 |
| 4.96 | 0.03 | 2.01 | 5.58 |
| 6.73 | 0.03 | 3.71 | 6.84 |
| 6.15 | 0.03 | 3.11 | 6.91 |
| 6.57 | 0.03 | 2.81 | 5.64 |
| 4.2 | 0.3 | 2.76 | 6.63 |
| 4.56 | 0.25 | 3.37 | 5.62 |
| 4.14 | 0.03 | 3.44 | 4.71 |
| 4.61 | 0.36 | 3.38 | 5.69 |
| 4.73 | 0.43 | 3.81 | 6.49 |
| 4.35 | 0.39 | 3.66 | 8.18 |
| 4.9 | 0.38 | 3.37 | 5.14 |
| 6.31 | 0.4 | 1.89 | 7.06 |
| 5.72 | 0.46 | 3.97 | 5.25 |
| 2.75 | 0.35 | 3.17 | 6.68 |
| 6.23 | 0.03 | 4.07 | 7.84 |
| 4.09 | 0.37 | 3.59 | 6.63 |
| 5.8 | 0.1 | 2.51 | 5.07 |
| 6.36 | 0.4 | 4.12 | 7.85 |
| 5.22 | 0.32 | 3.33 | 7.37 |
| 5.14 | 0.33 | 2.65 | 5.43 |
| 5.3 | 0.29 | 4.32 | 7.9 |
| 4.87 | 0.35 | 2.16 | 6.73 |
| 5.42 | 0.03 | 3.67 | 4.83 |
| 4.91 | 0.4 | 2.8 | 6.7 |
| 5.53 | 0.03 | 4.28 | 5.7 |
| 5.55 | 0.03 | 4.49 | 5.3 |
| 5.13 | 0.03 | 5.28 | 5.54 |
| 3.75 | 0.06 | 3.78 | 7.69 |
| 5.79 | 0.03 | 3.37 | 6.9 |
| 6.31 | 0.22 | 3.22 | 7.79 |
| 4.47 | 0.46 | 3.68 | 7.45 |
| 5.34 | 0.03 | 2.56 | 5.26 |
| 4.13 | 0.03 | 3.41 | 7.17 |
| 3.54 | 0.28 | 3.38 | 4.71 |
| 4.97 | 0.35 | 4.03 | 6.43 |
| 4.41 | 0.27 | 2.91 | 4.71 |
| 6.75 | 0.43 | 3.5 | 4.71 |
| 5.59 | 0.31 | 2.51 | 4.71 |
| 6.19 | 0.35 | 2.78 | 5.55 |
| 2.86 | 0.03 | 2.35 | 4.86 |
| 4.21 | 0.26 | 3.86 | 4.9 |
| 4.9 | 0.02 | 1.8 | 4.71 |
| 4.53 | 0.76 | 5.65 | 8.55 |

e3: Deformity rate (%)

| basic data 5+K2:M58 | NS3-12h | HF3-12h | m-HF -12h |
| --- | --- | --- | --- |
| 89.22 | 96.51 | 88.67 | 94.28 |
| 88.31 | 98.32 | 94.2 | 89.49 |
| 86.93 | 96.61 | 93.56 | 95.12 |
| 88.11 | 97.27 | 96.14 | 93.66 |
| 90.7 | 99.12 | 92.5 | 93.81 |
| 89.06 | 96.63 | 92.75 | 91.13 |
| 89.56 | 99.49 | 93.58 | 90.45 |
| 88.39 | 97.24 | 89.28 | 89.49 |
| 87.04 | 97.06 | 90.42 | 93.93 |
| 88.74 | 97.14 | 92.7 | 94.19 |
| 88.63 | 97.95 | 95.33 | 89.49 |
| 89.94 | 98.26 | 91.83 | 93.66 |
| 85.57 | 98.7 | 91.76 | 94.16 |
| 88.6 | 97.49 | 94.43 | 90.73 |
| 87.95 | 98.96 | 93.33 | 94.71 |
| 89.59 | 98.24 | 90.38 | 90.13 |
| 87.1 | 99.19 | 96.08 | 93.64 |
| 89.71 | 96.73 | 88.65 | 94.12 |
| 87.72 | 97.6 | 93.15 | 93.71 |
| 87.98 | 98.79 | 92.85 | 90.95 |
| 86.72 | 96.8 | 89.07 | 94.9 |
| 89.44 | 97.38 | 93.42 | 91.04 |
| 90.56 | 98.62 | 94.43 | 93.81 |
| 87.14 | 98.47 | 90.1 | 94.67 |
| 86.03 | 99.67 | 91.07 | 89.49 |
| 86.06 | 97.27 | 88.99 | 95.87 |
| 87.04 | 98.46 | 90.87 | 91.01 |
| 88.11 | 96.74 | 92.87 | 90.1 |
| 87.74 | 99.37 | 89.62 | 89.49 |
| 91.24 | 97.38 | 88.43 | 93.36 |
| 87.81 | 97.31 | 91.04 | 91.23 |
| 86.52 | 96.71 | 94.84 | 93.5 |
| 90.85 | 97.27 | 92.06 | 89.49 |
| 88.66 | 98.48 | 90.91 | 94.71 |
| 88.55 | 98.8 | 93.14 | 89.49 |
| 88.11 | 99.08 | 89.35 | 94.18 |
| 88.1 | 97.3 | 95.06 | 91.51 |
| 88.72 | 98.59 | 91.85 | 91.29 |
| 86.92 | 98.36 | 89.46 | 93.64 |
| 87.61 | 96.51 | 92.64 | 93.43 |
| 88.39 | 98.48 | 90.94 | 90.9 |
| 87.92 | 98.3 | 94.92 | 93.4 |
| 86.61 | 96.86 | 91.88 | 95.81 |
| 88.07 | 99.44 | 91.77 | 91.44 |
| 91.26 | 98.74 | 93.4 | 94.75 |
| 87.09 | 98.8 | 91.67 | 95.45 |
| 85.71 | 97.37 | 93.14 | 95.53 |
| 87.64 | 96.86 | 93.6 | 91.56 |
| 87.08 | 98.75 | 92.96 | 95.41 |
| 87.3 | 98.11 | 92.71 | 94.83 |
| 84.83 | 98.16 | 91.37 | 94.94 |
| 88.94 | 95.99 | 93.62 | 89.61 |
| 86.03 | 96.47 | 94 | 93.53 |
| 86.39 | 97.65 | 90.27 | 95.22 |
| 89.24 | 99.51 | 93.88 | 91.3 |
| 90.1 | 99.76 | 92.56 | 91.26 |
| 88.11 | 98.99 | 92.38 | 93.47 |
| 89.32 | 97.46 | 93.24 | 91.35 |
| 88.22 | 95.95 | 87.65 | 89.49 |
| 87.76 | 99.77 | 97.93 | 96.58 |

e4: Survival rate (%)

| basic data 5 | NS3-12h | HF3-12h | m-HF -12h |
| --- | --- | --- | --- |
| 67.62 | 4.49 | 54.84 | 56.01 |
| 79.29 | 6.76 | 53.87 | 53.28 |
| 68.98 | 4.45 | 54.55 | 54.33 |
| 71.16 | 6.25 | 48.67 | 52 |
| 69.62 | 6.14 | 48.5 | 61.08 |
| 55.25 | 5.03 | 55.94 | 56.77 |
| 74.49 | 5.8 | 52.14 | 58.12 |
| 74.95 | 4.19 | 51.92 | 54.2 |
| 75.37 | 6.76 | 51.22 | 59.47 |
| 71.58 | 4.39 | 53.65 | 58.56 |
| 63.77 | 6.76 | 53.66 | 58.66 |
| 62.33 | 6.52 | 53.71 | 55.82 |
| 69.44 | 6.14 | 54.09 | 59.89 |
| 72.14 | 4.96 | 54.97 | 59.22 |
| 70.29 | 4.73 | 55.34 | 58.33 |
| 62.1 | 6.44 | 54.79 | 55.99 |
| 71.1 | 3.9 | 52.1 | 60.24 |
| 82.05 | 3.87 | 50.61 | 61.55 |
| 68.97 | 5.15 | 54.01 | 56.03 |
| 69.58 | 4.8 | 54.54 | 56.49 |
| 63.13 | 6.5 | 51.77 | 60.08 |
| 62.62 | 5.9 | 54.09 | 57.62 |
| 69.13 | 5.03 | 55.94 | 52.44 |
| 71.33 | 5.22 | 54.3 | 60.64 |
| 72.19 | 6.46 | 55.22 | 55.83 |
| 64.38 | 4.76 | 53.63 | 54.22 |
| 71.31 | 4.38 | 50.4 | 58.03 |
| 63.33 | 5.07 | 51.79 | 59.67 |
| 66 | 3.59 | 55.93 | 52.39 |
| 68.7 | 6.76 | 50.53 | 53.83 |
| 64.78 | 6.03 | 54.32 | 60.8 |
| 58.74 | 5.78 | 54.21 | 51.68 |
| 67.28 | 5.85 | 53.97 | 55.64 |
| 69.63 | 4.12 | 55.94 | 52.67 |
| 68.64 | 4.68 | 51.04 | 53.68 |
| 66.62 | 5.74 | 52.22 | 56.03 |
| 72.05 | 4.62 | 55.12 | 57.72 |
| 72.96 | 6.52 | 54.57 | 57.91 |
| 66.59 | 4.84 | 52.65 | 53.36 |
| 69.43 | 4.8 | 55.5 | 53.14 |
| 77.31 | 3.72 | 52.72 | 61.55 |
| 66.58 | 6.41 | 53.73 | 60.54 |
| 58.99 | 4.13 | 52.13 | 54.67 |
| 60.33 | 6.48 | 54.43 | 60.51 |
| 76.88 | 4.38 | 52.34 | 59.2 |
| 69.62 | 6.22 | 51.64 | 61.55 |
| 73.02 | 6.6 | 55.94 | 51.56 |
| 70.31 | 4.73 | 55.52 | 58.8 |
| 69.22 | 5.19 | 54.31 | 61.55 |
| 61.37 | 6.65 | 51.29 | 54.6 |
| 74.51 | 4.67 | 54.96 | 61.55 |
| 66.16 | 6.38 | 52.72 | 53.51 |
| 70.52 | 4.6 | 51.82 | 59.11 |
| 72.13 | 5.92 | 47.5 | 59.45 |
| 69.82 | 6.11 | 51.36 | 61.55 |
| 65.81 | 3.61 | 55.54 | 60.3 |
| 76.54 | 4.23 | 52.41 | 58.03 |
| 71.91 | 4.7 | 52.55 | 55.01 |
| 77.17 | 3.23 | 47.2 | 50.7 |
| 74.59 | 6.76 | 55.94 | 61.55 |

e5: Sperm DNA fragmentation rate (%)

| basic data 5+U2:X62 | NS3-12h | HF3-12h | m-HF -12h |
| --- | --- | --- | --- |
| 16.9 | 14.92 | 19.78 | 15.76 |
| 16.08 | 20.04 | 19.55 | 17.94 |
| 16.5 | 22.6 | 16.27 | 18.79 |
| 16.91 | 16.91 | 18.13 | 16.78 |
| 15.63 | 20.55 | 19.58 | 16.15 |
| 14.75 | 18.66 | 18.19 | 16.35 |
| 15.41 | 19.4 | 18.47 | 18.21 |
| 16.14 | 17.98 | 14.44 | 19 |
| 20.1 | 23.31 | 15.26 | 16.84 |
| 16.69 | 23.57 | 13.51 | 16.4 |
| 19.09 | 22.45 | 19.84 | 15.7 |
| 16.82 | 20.18 | 20 | 18.18 |
| 14.89 | 18.89 | 17.11 | 17.96 |
| 17.89 | 16.67 | 17.83 | 17.73 |
| 15.86 | 22.19 | 19.87 | 18.47 |
| 18.67 | 16.86 | 18.76 | 17.88 |
| 15.99 | 21.77 | 13.71 | 16.44 |
| 17.2 | 16.77 | 20.26 | 19 |
| 17.59 | 21.06 | 16.71 | 16.21 |
| 16.99 | 21.12 | 17.6 | 16.62 |
| 18.75 | 18.44 | 18.49 | 18.66 |
| 15.21 | 15.43 | 19.84 | 16.08 |
| 18.14 | 20.89 | 18.69 | 16.52 |
| 17.93 | 18.42 | 16.52 | 15.78 |
| 15.07 | 15.7 | 17.72 | 17.67 |
| 16.97 | 23.52 | 14.33 | 17.93 |
| 15.24 | 19.08 | 14.24 | 15.7 |
| 17.3 | 21.78 | 17.12 | 18.23 |
| 19.18 | 23.08 | 15.69 | 16.59 |
| 15.83 | 15.05 | 16.05 | 16.12 |
| 17.03 | 16.44 | 20.04 | 17.91 |
| 16.94 | 22.62 | 17.81 | 17.63 |
| 16.13 | 21.96 | 19.47 | 16.9 |
| 17.47 | 16.62 | 13.51 | 19 |
| 18.42 | 15.57 | 16.51 | 16.9 |
| 15.31 | 16.54 | 17.82 | 17.82 |
| 16.69 | 16.73 | 15.97 | 16.1 |
| 18.43 | 19.49 | 16.1 | 15.71 |
| 15.62 | 22.74 | 15.85 | 16.78 |
| 18.15 | 21.06 | 15.58 | 19 |
| 20.47 | 20.82 | 18.94 | 18.77 |
| 18.53 | 22.36 | 20.26 | 16.8 |
| 17.62 | 18.82 | 18.08 | 19 |
| 17.73 | 14.74 | 18.39 | 18.06 |
| 15.62 | 15.91 | 19.06 | 15.91 |
| 17.02 | 20.43 | 15.95 | 16.86 |
| 16.36 | 18.24 | 15.82 | 18.66 |
| 17.86 | 17.13 | 19.66 | 18.21 |
| 15.65 | 20.61 | 16.3 | 18.09 |
| 15.75 | 21.81 | 16.57 | 18.52 |
| 17.37 | 20.74 | 16.87 | 18.63 |
| 20.28 | 18.7 | 14.73 | 15.7 |
| 18.15 | 18.31 | 14.71 | 17.96 |
| 15.26 | 21.72 | 19.04 | 16.6 |
| 19.7 | 20.94 | 15.93 | 16.79 |
| 17.81 | 18.47 | 18.95 | 15.7 |
| 19.58 | 19.53 | 20.26 | 15.96 |
| 15.46 | 17.91 | 17.12 | 18.55 |
| 16.88 | 14.07 | 13.39 | 15.7 |
| 16.1 | 23.58 | 20.26 | 19 |
