# Supplementary material for: The effects of L-carnitine and fructose in improved Ham’s F10 on sperm culture in idiopathic severe asthenospermia within 24h
Source: PLoS One. 2025 Feb 10;20(2):e0306235. doi: 10.1371/journal.pone.0306235 (PMC11809793; doi:10.1371/journal.pone.0306235)
Supplement: S6 File — (DOCX) [file pone.0306235.s006.docx]

f1: Percentage of forward motility of sperm (%)

| basic data 6 | NS3-24h | HF3-24h | m-HF -24h |
| --- | --- | --- | --- |
| 7.12 | 2.31 | 4.83 | 5.43 |
| 6.99 | 1.28 | 3.93 | 5.5 |
| 6.31 | 0.72 | 4.93 | 9.35 |
| 6.66 | 0.4 | 3.83 | 5.4 |
| 8.51 | 0.48 | 3.97 | 6.12 |
| 6.86 | 0.28 | 3.1 | 7.31 |
| 6.78 | 0.8 | 2.21 | 6.91 |
| 7.52 | 0.15 | 2.89 | 8.68 |
| 4.47 | 1.06 | 3.44 | 5.74 |
| 8.4 | 0.14 | 4.03 | 8.07 |
| 4.86 | 0.53 | 5.68 | 3.15 |
| 6.05 | 4.65 | 0.63 | 7.6 |
| 5.28 | 2.59 | 2.79 | 7.3 |
| 5.35 | 2.18 | 4.61 | 2.46 |
| 7.19 | 0.09 | 2.13 | 5.47 |
| 7.4 | 0.46 | 3.43 | 6.19 |
| 6.78 | 1.34 | 4.38 | 4.5 |
| 8.04 | 4.33 | 1.58 | 5.93 |
| 6.39 | 1.54 | 3.12 | 6.6 |
| 6.16 | 0.21 | 3.18 | 4.59 |
| 7.61 | 0.21 | 4.16 | 8.1 |
| 7.14 | 0.27 | 4.93 | 6.87 |
| 7.77 | 0.21 | 4.08 | 5.36 |
| 7.31 | 0.21 | 3.8 | 5.47 |
| 7.91 | 0.24 | 4.2 | 5.79 |
| 5.91 | 2.86 | 1.82 | 5.42 |
| 7.53 | 1.65 | 4.93 | 5.09 |
| 5.62 | 0.21 | 3.86 | 5.78 |
| 7.15 | 0.25 | 4.68 | 6.66 |
| 4.68 | 0.21 | 3.89 | 4.59 |
| 8.87 | 0.21 | 3.96 | 6.72 |
| 7.33 | 0.21 | 4.05 | 4.07 |
| 7.21 | 0.57 | 1.85 | 6.54 |
| 5.93 | 2.27 | 2.66 | 6.45 |
| 7.69 | 0.21 | 4.27 | 6.96 |
| 8.74 | 2.09 | 2.44 | 3.93 |
| 6.75 | 3.89 | 4.4 | 6.8 |
| 5.4 | 2.21 | 4.36 | 3.78 |
| 5.89 | 2.18 | 4.39 | 5.72 |
| 7.53 | 1.87 | 4.93 | 4.59 |
| 6.5 | 3.16 | 3.84 | 3.98 |
| 8.2 | 2.96 | 2.2 | 4.39 |
| 7.16 | 0.21 | 3.75 | 5.79 |
| 6.93 | 2.28 | 4.93 | 7.79 |
| 5.6 | 0.21 | 3.91 | 7.85 |
| 7.81 | 0.33 | 1.21 | 8.26 |
| 5.31 | 0.21 | 0.99 | 4.41 |
| 7.86 | 0.21 | 2.34 | 8.2 |
| 7.08 | 2.86 | 4.85 | 8.49 |
| 7.88 | 0.21 | 2.63 | 8.66 |
| 6.56 | 2.45 | 2.41 | 8.68 |
| 7 | 0.21 | 1.99 | 5.01 |
| 5.89 | 0.39 | 3.89 | 7.26 |
| 7.42 | 0.21 | 3.83 | 4.89 |
| 7.4 | 3.23 | 3.92 | 8.68 |
| 5.96 | 3.22 | 2.63 | 3.69 |
| 8.19 | 1.95 | 2.32 | 3.91 |
| 7.5 | 0.21 | 2.53 | 8.14 |
| 6.67 | 0.21 | 0.63 | 3.15 |
| 5.3 | 4.65 | 4.93 | 8.68 |

f2: Percentage of non-forward motile sperm (%)

| basic data 6 | NS3-24h | HF3-24h | m-HF -24h |
| --- | --- | --- | --- |
| 6.15 | 0.22 | 1.32 | 7.2 |
| 6.73 | 0.03 | 2.62 | 5.06 |
| 4.96 | 0.13 | 2.95 | 4.39 |
| 5.54 | 0.05 | 2.26 | 6.03 |
| 6 | 0.12 | 1.78 | 3.25 |
| 5.65 | 0.1 | 1.87 | 5.31 |
| 3.34 | 0.05 | 2.54 | 5.62 |
| 2.74 | 3.51 | 1.73 | 4.64 |
| 6.65 | 0.12 | 1.99 | 1.67 |
| 5.07 | 0.14 | 1.67 | 3.9 |
| 4.51 | 0.03 | 1.98 | 4.52 |
| 5.61 | 0.15 | 2.71 | 5.97 |
| 4.5 | 0.13 | 2.74 | 6.79 |
| 5.26 | 0.11 | 2.47 | 5.87 |
| 6 | 0.35 | 2.7 | 3.33 |
| 5.45 | 3.38 | 2.16 | 4.87 |
| 3.87 | 0.15 | 2.5 | 4.84 |
| 3.98 | 0.03 | 1.57 | 1.19 |
| 6.49 | 0.27 | 2.73 | 4.18 |
| 4.06 | 0.02 | 2.8 | 4.08 |

f3: Deformity rate (%)

| basic data 6 | NS3-24h | HF3-24h | m-HF -24h |
| --- | --- | --- | --- |
| 89.22 | 100.2 | 97.47 | 91.25 |
| 88.31 | 97.98 | 98.41 | 96.72 |
| 86.93 | 97.98 | 93.5 | 92.56 |
| 88.11 | 97.81 | 96.46 | 95.56 |
| 90.7 | 97.98 | 95.12 | 95.49 |
| 89.06 | 97.79 | 96.26 | 90.92 |
| 89.56 | 97.39 | 96.55 | 91.37 |
| 88.39 | 95.74 | 93.14 | 97.45 |
| 87.04 | 97.98 | 93.77 | 93.54 |
| 88.74 | 96.41 | 99.05 | 97.45 |
| 88.63 | 98.49 | 95.05 | 95.74 |
| 89.94 | 96.65 | 97.76 | 97.45 |
| 85.57 | 97.71 | 94.03 | 91.96 |
| 88.6 | 97.98 | 90.64 | 96.78 |
| 87.95 | 99.12 | 91.86 | 96.02 |
| 89.59 | 99.69 | 97.63 | 91.83 |
| 87.1 | 98.54 | 98.28 | 91.81 |
| 89.71 | 98.08 | 90.5 | 92.77 |
| 87.72 | 97.98 | 96.33 | 92.13 |
| 87.98 | 97.98 | 91.77 | 91.77 |
| 86.72 | 99.25 | 91.37 | 95.38 |
| 89.44 | 97.98 | 95.32 | 97.45 |
| 90.56 | 99.29 | 96.77 | 92.5 |
| 87.14 | 98.85 | 95.76 | 97.45 |
| 86.03 | 96.44 | 95.94 | 92.25 |
| 86.06 | 96.67 | 95.78 | 97.14 |
| 87.04 | 97.95 | 91.97 | 95.21 |
| 88.11 | 97.58 | 96.59 | 95.63 |
| 87.74 | 99.12 | 95.12 | 89.78 |
| 91.24 | 99.43 | 93.77 | 95.43 |
| 87.81 | 98.31 | 92.83 | 95.78 |
| 86.52 | 96.28 | 99.38 | 91.18 |
| 90.85 | 96.55 | 96.2 | 97.45 |
| 88.66 | 98.89 | 98.65 | 96.09 |
| 88.55 | 98.12 | 99.15 | 95.39 |
| 88.11 | 97.59 | 94.29 | 95.63 |
| 88.1 | 99.37 | 97.65 | 95.85 |
| 88.72 | 97.86 | 95.97 | 88.92 |
| 86.92 | 96.7 | 96.79 | 96.72 |
| 87.61 | 99.18 | 96.41 | 95.48 |
| 88.39 | 97.96 | 93.65 | 95.32 |
| 87.92 | 97.37 | 94.76 | 90.33 |
| 86.61 | 97.36 | 92.47 | 92.83 |
| 88.07 | 98.47 | 92.98 | 89.42 |
| 91.26 | 96.55 | 97.87 | 97.45 |
| 87.09 | 97.9 | 95.43 | 95.42 |
| 85.71 | 97.98 | 98.99 | 97.45 |
| 87.64 | 97.65 | 93.99 | 97.45 |
| 87.08 | 98.46 | 93.78 | 92.7 |
| 87.3 | 98.75 | 98.04 | 95.59 |
| 84.83 | 98.68 | 95.23 | 96.8 |
| 88.94 | 97.98 | 93.01 | 96.49 |
| 86.03 | 97.98 | 92.57 | 95.22 |
| 86.39 | 97.98 | 96.64 | 92.86 |
| 89.24 | 98.26 | 94.71 | 96.48 |
| 90.1 | 98.69 | 94.25 | 91.67 |
| 88.11 | 96.58 | 95.38 | 90.34 |
| 89.32 | 97.43 | 94.68 | 95.26 |
| 88.3 | 95.74 | 89.91 | 88.1 |
| 87.76 | 100.2 | 102.5 | 97.45 |

f4: Survival rate (%)

| basic data 6 | NS3-24h | HF3-24h | m-HF -24h |
| --- | --- | --- | --- |
| 67.62 | 7.05 | 29.68 | 37.84 |
| 79.29 | 4.5 | 30.17 | 37.96 |
| 68.98 | 4.84 | 31.64 | 37.18 |
| 71.16 | 4.75 | 28.66 | 35.83 |
| 69.62 | 6.5 | 29.42 | 35.34 |
| 55.25 | 6.29 | 34.44 | 37.75 |
| 74.49 | 4.76 | 30.81 | 32.05 |
| 74.95 | 5.92 | 30.92 | 33.51 |
| 75.37 | 4.57 | 33.26 | 35.37 |
| 71.58 | 4.71 | 34.31 | 33.19 |
| 63.77 | 7.05 | 30.39 | 37.42 |
| 62.33 | 4.04 | 31.16 | 32.71 |
| 69.44 | 6.17 | 32.98 | 36.21 |
| 72.14 | 5.88 | 29.92 | 33.09 |
| 70.29 | 3.63 | 35.01 | 35.38 |
| 62.1 | 3.62 | 28.85 | 32.35 |
| 71.1 | 4.88 | 28.77 | 31.71 |
| 82.05 | 5.84 | 28.9 | 35.65 |
| 68.97 | 3.81 | 28.79 | 35.78 |
| 69.58 | 3.53 | 34.64 | 31.92 |
| 63.13 | 4.56 | 30.63 | 33.55 |
| 62.62 | 5.68 | 28.44 | 37.96 |
| 69.13 | 5.65 | 29.96 | 32.34 |
| 71.33 | 7.05 | 30.54 | 35.66 |
| 72.19 | 3.52 | 33.21 | 35.07 |
| 64.38 | 4.59 | 35.28 | 31.14 |
| 71.31 | 4.95 | 35.28 | 35.8 |
| 63.33 | 6.46 | 34.75 | 37.96 |
| 66 | 4.96 | 32.38 | 32.45 |
| 68.7 | 4.05 | 34.59 | 33.26 |
| 64.78 | 6.07 | 30.68 | 34.09 |
| 58.74 | 3.84 | 32.62 | 37.53 |
| 67.28 | 4.79 | 32.18 | 37.19 |
| 69.63 | 7.05 | 33.55 | 35.79 |
| 68.64 | 5.85 | 31.18 | 32.81 |
| 66.62 | 3.73 | 34.81 | 35.13 |
| 72.05 | 6.72 | 29.04 | 30.89 |
| 72.96 | 4.35 | 34.33 | 33.1 |
| 66.59 | 4.46 | 29.89 | 33.55 |
| 69.43 | 4.78 | 33.13 | 33.06 |
| 77.31 | 3.58 | 32.26 | 31.67 |
| 66.58 | 4.72 | 33.48 | 36.62 |
| 58.99 | 5.57 | 30.14 | 36.3 |
| 60.33 | 7.05 | 32.96 | 37.47 |
| 76.88 | 6.57 | 29.6 | 37.96 |
| 69.62 | 4.39 | 32.29 | 32.98 |
| 73.02 | 5.58 | 30.75 | 31.8 |
| 70.31 | 5.84 | 30.24 | 33.31 |
| 69.22 | 5.02 | 28.52 | 36.41 |
| 61.37 | 6.66 | 30.31 | 35.49 |
| 74.51 | 4.49 | 32.86 | 33.01 |
| 66.16 | 4.83 | 29.66 | 33.36 |
| 70.52 | 6.37 | 33.21 | 35.33 |
| 72.13 | 4.62 | 34.03 | 35.34 |
| 69.82 | 3.73 | 30.79 | 33.57 |
| 65.81 | 6.13 | 28.68 | 31 |
| 76.54 | 5.69 | 34.32 | 30.86 |
| 71.91 | 5.6 | 31.13 | 37.24 |
| 77.17 | 3.37 | 28.4 | 30.85 |
| 74.59 | 7.05 | 35.28 | 37.96 |

f5: Sperm DNA fragmentation rate (%)

| basic data 6 | NS3-24h | HF3-24h | m-HF -24h |
| --- | --- | --- | --- |
| 16.9 | 21.01 | 22.75 | 18 |
| 16.08 | 23.63 | 22.38 | 17 |
| 16.5 | 23.24 | 22.78 | 20 |
| 16.91 | 23.68 | 20.29 | 20 |
| 15.63 | 25.52 | 17.04 | 20 |
| 14.75 | 22.58 | 25.28 | 20 |
| 15.41 | 28.81 | 21.39 | 20 |
| 16.14 | 27.21 | 21.32 | 19 |
| 20.1 | 27.22 | 20.23 | 18 |
| 16.69 | 27.69 | 22.03 | 20 |
| 19.09 | 24.99 | 24.03 | 20 |
| 16.82 | 23.12 | 21.58 | 20 |
| 14.89 | 25.13 | 20.31 | 19 |
| 17.89 | 19.16 | 24.07 | 18 |
| 15.86 | 23.25 | 22.9 | 20 |
| 18.67 | 22.08 | 18.08 | 20 |
| 15.99 | 24.93 | 17.73 | 19 |
| 17.2 | 22.13 | 17.49 | 18 |
| 17.59 | 25.81 | 18.57 | 18 |
| 16.99 | 27.22 | 16.4 | 18 |
| 18.75 | 29.02 | 23.52 | 17 |
| 15.21 | 21.36 | 18.05 | 18 |
| 18.14 | 21.32 | 23.73 | 20 |
| 17.93 | 26.35 | 22.14 | 20 |
| 15.07 | 23.03 | 20.32 | 20 |
| 16.97 | 25.44 | 23.9 | 19 |
| 15.24 | 25.43 | 18.22 | 17 |
| 17.3 | 21.39 | 17.51 | 16 |
| 19.18 | 23.81 | 22.36 | 18 |
| 15.83 | 21.67 | 15.88 | 20 |
| 17.03 | 20.54 | 20.03 | 18 |
| 16.94 | 22.42 | 19.82 | 20 |
| 16.13 | 28.24 | 21.78 | 17 |
| 17.47 | 22.9 | 18.22 | 16 |
| 18.42 | 29.21 | 22.91 | 18 |
| 15.31 | 25.78 | 18.74 | 19 |
| 16.69 | 27.18 | 21.83 | 17 |
| 18.43 | 20.55 | 25.28 | 17 |
| 15.62 | 26.65 | 20.96 | 17 |
| 18.15 | 26.13 | 24.09 | 18 |
| 20.47 | 22.61 | 23.62 | 16 |
| 18.53 | 23.8 | 23.4 | 19 |
| 17.62 | 27.57 | 17.09 | 16 |
| 17.73 | 23.24 | 19.86 | 18 |
| 15.62 | 22.65 | 16.36 | 18 |
| 17.02 | 28.73 | 19.3 | 20 |
| 16.36 | 26.44 | 19.28 | 19 |
| 17.86 | 26.69 | 22.01 | 18 |
| 15.65 | 25.62 | 21.4 | 18 |
| 15.75 | 21.05 | 23.04 | 20 |
| 17.37 | 23.14 | 16.84 | 17 |
| 20.28 | 23.89 | 23.69 | 19 |
| 18.15 | 21.75 | 15.55 | 17 |
| 15.26 | 27.42 | 20.12 | 18 |
| 19.7 | 22.63 | 19.97 | 20 |
| 17.81 | 22.14 | 17.21 | 19 |
| 19.58 | 23.56 | 22.5 | 18 |
| 15.46 | 23.77 | 22.38 | 20 |
| 16.88 | 19.16 | 15.06 | 16 |
| 16.1 | 29.21 | 25.28 | 21 |
