# Supplementary material for: The effects of L-carnitine and fructose in improved Ham’s F10 on sperm culture in idiopathic severe asthenospermia within 24h
Source: PLoS One. 2025 Feb 10;20(2):e0306235. doi: 10.1371/journal.pone.0306235 (PMC11809793; doi:10.1371/journal.pone.0306235)
Supplement: Attachment — Submitted filename: renamed_70cc5.docx [file pone.0306235.s007.docx]

**Author’s Response to Reviewers‘ Comments**

Ms. No.: PONE-D-24-23439

Title: The effects of L-carnitine and fructose in improved Ham's F10 on sperm culture in idiopathic severe asthenospermia within 24h

Journal: PLOS ONE

Editor and Reviewer comments:

Especially the points from Reviewer #2 that the author should introduce the definition, occurrence, ART outcome, ways to improve ART outcome in men with idiopathic severe asthenospermia, as well as how to recruit these patients.

Reply:

According to WHO laboratory manual for the examination and processing of human semen (5th Edition), asthenospermia is defined as progressive motility < 32% (abstinence for 2-7 days). Idiopathic asthenospermia is understood as a form of asthenospermia with unexplained and unknown etiology. However, the definition of severe asthenozoospermia is different in different race. Severe asthenozoospermia refers to patient with 1 ~ 10% (China) or < 20% (Spain) progressive motility. Therefore, idiopathic severe asthenospermia refers to Chinese patient with 1 ~ 10% progressive motility with [undiscovered](javascript:%20void(0)) etiology in this study. Although conventional in vitro fertilization (IVF) or intracytoplasmic sperm injection (ICSI) can lead to successful pregnancy in couples with only male infertility, progressive motility has a certain influence on the outcome of ART. Therefore, using sperm culture medium to improve sperm motility as much as possible is helpful to improve ART outcome. The above detailed explanations are supported by updated literature.

Ethics approval by the Human Ethics Committee of the 940 Hospital (IRB approval No. 2016KYLL086). And the use of this experimental semen specimens and the experimental protocol were agreed. The clinical trial number: 2016CLT-U002.We informed each participant the details of the study: We need to collect some semen amples from you, and only use the remaining semen samples from the routine semen test. The sample is only used in this study and will not affect routine treatment or diagnosis. We obtained written informed consent forms from each participants included in this study.

We recruited 60 patients with idiopathic severe asthenozoospermia (the criteria was that forward motility of sperm is 1 ~ 10% and with unknown etiology), aged 24-35 (26.5±4.31) years, participated in this study at the Urology Center of the 940th Hospital, from September 2017 to August 2018. We informed each participant the details of the study: We need to collect some semen samples from you, and only use the remaining semen samples from the routine semen test. The sample is only used in this study and will not affect routine treatment or diagnosis. Informed consent forms were obtained from the patients included in this study.

Reply to Journal Requirements:

1. We update the manuscript to meet PLOS ONE's style requirements, including those for file naming.

2. We provide additional details regarding participant consent, which is stated in the ethics statement in the Methods and online submission information. Informed consent forms were obtained from the patients included in this study. We upload the Informed consent for clinical participant.

3. Dehui Chang and Bin Zhang are the funders of above funds. Dehui Chang contributed to the whole project development. Bin Zhang contributed to carrying out the literature review, the language translation, grammar checking, and manuscript writing.

4. We confirm at this time the submission contains all raw data required to replicate the results of this study. We submit and upload the following data:

- The values behind the means, standard deviations and other measures reported;

- The values used to build graphs;

- The points extracted from images for analysis.

- Some minor data errors have been corrected, and the icons has been corrected..

5. By using PACE digital diagnostic tool, We transformed and uploaded the figure files.

Reply to Reviewer #1:

1. Thank you for your review. We standarded the writing and corrected mg/mL to mg/ml.

2. When the table spreads, we have adjusted the table to one page or used a continuation table.

3. According to your suggestion, we rewrote the abbreviations of the group names as follows:

A1: NS, B1: L4, C1: L8, D1: L12, E1: L16, F1:HF.

A2: NS, B2: F0.125, C2: F0.250, D2: F0.375, E2: F0.500, F2:HF.

A3:NS (NS--30min, NS3-1h, NS3-12h, and NS3-24h), B3:HF (HF3-30min, HF3-1h, HF3-12h, and HF3-24h), C3: m-HF (m-HF -30min, m-HF -1h, m-HF -12h, and m-HF -24h).

4. We corrected all writing errors in the text.

Reply to Reviewer #2:

1. In the introduction, we have introduced the definition, occurrence, ART outcome and methods to improve ART outcome in patients with idiopathic azoospermia and idiopathic severe asthenospermia in detail. In the discussion, we emphasized the significance of this study for improving sperm motility in patients with idiopathic asthenospermia in vitro.

2. Because the contents of table 1 and table 2 are duplicated, we deleted table 1 and kept table 2 and 3. We put some figures into the supplementary data, and kept important results in the main document.

3. This paper has been significantly revises. Please refer to a website to help modify the language of the article. The editor’s native language is English. Thank you!

Thank you for your help and support!

Your sincerely yours.

Bin Zhang

2024-09-05
