# Supplementary material for: The effects of L-carnitine and fructose in improved Ham’s F10 on sperm culture in idiopathic severe asthenospermia within 24h
Source: PLoS One. 2025 Feb 10;20(2):e0306235. doi: 10.1371/journal.pone.0306235 (PMC11809793; doi:10.1371/journal.pone.0306235)
Supplement: Attachment — Submitted filename: renamed_52410.docx [file pone.0306235.s008.docx]

**Author’s Response to Reviewers‘ Comments**

Ms. No.: PONE-D-24-23439R1

Title: The effects of L-carnitine and fructose in improved Ham's F10 on sperm culture in idiopathic severe asthenospermia within 24h

Journal: PLOS ONE

Editor and Reviewer comments:

Especially the points from Reviewer #2 that deletion of this paragraph in the INTRODUCTION part for the azoospermia is suggested since this work is not related to the azoospermia at all.

Reply to Journal Requirements:

1. At present, we have no laboratory protocols, and we will strengthen our work in this area. Thank you for your proposal.

2. The references have been revised and adjusted. The DOI and PMID code is supplemented in the reference [6], [19], [20], [21], and [22]. The original reference [18] was adjusted to reference [7], and references [7-17] was adjusted to reference [8-18].

3. We reviewed the reference list and ensure that it is complete and correct, in which all cited papers were searched on Pubmed and have not been retracted.

Reply to Reviewer #2 about “6. Review Comments to the Author”:

We deleted the paragraph in the INTRODUCTION part for the azoospermia since this work is not related to the azoospermia at all, according to the suggestion. The reference for this paragraph is the original reference [18]. The original reference [18] was about the study between male infertility and precision medicine and systems proteomics, so it was adjusted to the paragraph: “Male infertility is mainly manifested as azoospermia” in the INTRODUCTION part (line 75-76). For this reason, we adjusted the original reference [18] to reference [7], and references [7-17] to reference [8-18].

Thank you for your help and support!

Your sincerely yours.

Bin Zhang

2024-11-16
